# Comparative Genomics and Metabolic Analysis Reveals Peculiar Characteristics of *Rhodococcus opacus* Strain M213 Particularly for Naphthalene Degradation

**DOI:** 10.1371/journal.pone.0161032

**Published:** 2016-08-17

**Authors:** Ashish Pathak, Ashvini Chauhan, Jochen Blom, Karl J. Indest, Carina M. Jung, Paul Stothard, Gopal Bera, Stefan J. Green, Andrew Ogram

**Affiliations:** 1 School of the Environment, Florida A&M University, Tallahassee, Florida, United States of America; 2 Bioinformatics and Systems Biology, Justus-Liebig-University Giessen, Giessen, Germany; 3 Environmental Processes Branch, United States Army Engineer Research and Development Center, Vicksburg, Mississippi, United States of America; 4 Department of Agricultural, Food and Nutritional Science, University of Alberta, Edmonton, Canada; 5 Geochemical and Environmental Research Group, Texas A&M University, College Station, Texas, United States of America; 6 DNA Services Facility, University of Illinois at Chicago, Chicago, Illinois, United States of America; 7 Soil and Water Science Department, University of Florida, Gainesville, Florida, United States of America; Universite Paris-Sud, FRANCE

## Abstract

The genome of *Rhodococcus opacus* strain M213, isolated from a fuel-oil contaminated soil, was sequenced and annotated which revealed a genome size of 9,194,165 bp encoding 8680 putative genes and a G+C content of 66.72%. Among the protein coding genes, 71.77% were annotated as clusters of orthologous groups of proteins (COGs); 55% of the COGs were present as paralog clusters. Pulsed field gel electrophoresis (PFGE) analysis of M213 revealed the presence of three different sized replicons- a circular chromosome and two megaplasmids (pNUO1 and pNUO2) estimated to be of 750Kb 350Kb in size, respectively. Conversely, using an alternative approach of optical mapping, the plasmid replicons appeared as a circular ~1.2 Mb megaplasmid and a linear, ~0.7 Mb megaplasmid. Genome-wide comparative analysis of M213 with a cohort of sequenced *Rhodococcus* species revealed low syntenic affiliation with other *R*. *opacus* species including strains B4 and PD630. Conversely, a closer affiliation of M213, at the functional (COG) level, was observed with the catabolically versatile *R*. *jostii* strain RHA1 and other Rhodococcii such as *R*. *wratislaviensis* strain IFP 2016, *R*. *imtechensis* strain RKJ300, *Rhodococcus* sp. strain JVH1, and *Rhodococcus* sp. strain DK17, respectively. An in-depth, genome-wide comparison between these functional relatives revealed 971 unique genes in M213 representing 11% of its total genome; many associating with catabolic functions. Of major interest was the identification of as many as 154 genomic islands (GEIs), many with duplicated catabolic genes, in particular for PAHs; a trait that was confirmed by PCR-based identification of naphthalene dioxygenase (NDO) as a representative gene, across PFGE-resolved replicons of strain M213. Interestingly, several plasmid/GEI-encoded genes, that likely participate in degrading naphthalene (NAP) via a peculiar pathway, were also identified in strain M213 using a combination of bioinformatics, metabolic analysis and gene expression measurements of selected catabolic genes by RT-PCR. Taken together, this study provides a comprehensive understanding of the genome plasticity and ecological competitiveness of strain M213 likely facilitated by horizontal gene transfer (HGT), bacteriophage attacks and genomic reshuffling- aspects that continue to be understudied and thus poorly understood, in particular for the soil-borne Rhodococcii.

## Introduction

Naphthalene (NAP) is the simplest polycyclic aromatic hydrocarbons (PAHs), but due to high volatility, it is found widely distributed in the environment as a pollutant. PAHs, including NAP, are introduced into the environment mainly from industrial emissions, burning of plant biomass, combustion of gasoline and oil, tobacco smoke, and use of fumigants and deodorizers [1]. Thus, the ubiquitous environmental distribution of PAHs is a concern mainly owing to their carcinogenic and toxic impacts [2,3]. Once released into the environment, the biological fate of NAP is mineralization into CO_2_ and H_2_O via microbially-mediated processes [4]; driven mainly as a function of NAP bioavailability and the presence of biodegradative microorganisms.

A vast majority of the NAP metabolizing bacteria characterized to date are Gram-negative; primarily represented by the pseudomonads [5], which grow rapidly in the presence of high concentrations of nutrients, such as those present in standard laboratory enrichments amended with PAHs. Other bacterial species, including many soil-borne actinomycetes, exhibit lower Km (Monod growth coefficient) and lower maximum growth rates for some PAHs suggesting adaptation to low nutrient concentrations, such that actinomycetes potentially occupy environmental niches other than those occupied by pseudomonads. Not surprisingly, NAP degradative pathways and genes from the Actinobacterial genus- *Rhodococcus*, are generally not closely related to their counterparts from *Pseudomonas* [6–8]. From an environmental standpoint, Rhodococcii continue to be understudied, relative to the well-studied Pseduomonads, despite their strong potential for biodegradation of toxic pollutants as well as production of industrial biochemicals and biofuels [7–9]. Such attractive features of Rhodococcii are mainly attributed to their high metabolic versatility [10–12].

This metabolic strength typically found in *Rhodococcus* spp., seems to have arisen from the metabolism of soil organic carbon; the primary carbon and energy source for soil heterotrophic microbiota and the ability to degrade a vast majority of hydrocarbon pollutants. Hydrocarbons are metabolized by Rhodococcii via several peripheral pathways that channel catabolic substrates into a smaller pool of common intermediates, such as catechol, protocatechuate, and gentisate [5], which are eventually funnelled into the TCA cycle supporting bacterial growth and metabolism.

Among the previously studied NAP catabolic pathways, the most widespread pathway is via the conversion of NAP into salicylate (SAL) as a metabolic intermediate (http://eawag-bbd.ethz.ch/naph/naph_image_map.html), as is mostly the case with the Pseudomonads. Conversely, some Gram-positive bacteria, to include *Bacillus*, *Geobacillus* and *Mycobacterium*, do not use the standard SAL pathway and degrade NAP via the formation of *o*-phthalate (OPA) [13–15]. More specifically amongst Rhodococcii, it has been demonstrated that strains NCIMB 12038 [16], B4 [17] and R7 [18] degrade NAP via the formation of gentisate as a metabolic intermediate, whereas strains P200 and P400 degrade NAP via catechol [19]. Notably, just before this study was communicated, it was shown that *R*. *ruber* strain OA1 generates both SAL and OPA as metabolic intermediates during the degradation of NAP with protocatechuate as the terminal product prior to ring cleavage [20]. In fact, OPA has been previously demonstrated as a metabolic intermediate during NAP degradation, but only in thermophilic bacteria such as *Bacillus thermoleovorans* [13] and *Geobacillus* sp. G27 [15]. Therefore, considerable interest exists on the pathways by which NAP is microbially degraded because such studies can provide recommendations on appropriate environmental restoration activities to follow for better management of PAH-impacted environments. As an example, it has been demonstrated that the expression of biodegradative genes can be maintained at an optimum level by salicylate amendment, resulting in enhanced in-situ biodegradation of PAHs [21–23]–an approach that seems to hold great value in rehabilitation of PAH polluted environments.

At the genomic level, NAP degradative genes in Rhodococcii have been mapped onto different operons that are inducible by NAP, SAL or both [6; 16, 18]. Interestingly, a NAP degradative pathway in *Rhodococcus* strain TFB [24] has been demonstrated in which at least three sets of enzymes were found to be differentially regulated and inducible by NAP or tetralin but not SAL, the salicylaldehyde dehydrogenase inducible by NAP but not by SAL or tetralin, and the Nag enzymes that were induced by SAL but not by NAP or tetralin. These findings represent a paradigm shift on the assembly of a biodegradation pathway by way of recruiting genes belonging to different regulons, thus facilitating survival of the host bacterium in polluted environments.

To gain a better understanding on the repertoire of catabolic potential of actinomycetes, whole genome sequence comparisons were drawn between *R*. *opacus* strain M213- an isolate that we previously obtained from a fuel-oil contaminated soil [25] and a cohort of *Rhodococcus* species for which genome sequences are available in the Integrated Microbial Genomes (IMG) database [26]. Our previous analyses of strain M213 provided a comprehensive understanding on the range of catabolic genes encoded by this strain, specifically for the degradation of PAHs [naphthalene, phenanthrene, anthracene, benzo(a)pyrene] and a variety of halogenated aromatics and aromatic hydrocarbons [27]. Because genome-wide variations between different bacterial strains, especially the variable part of the genome including their mobile elements and genomic islands, have been shown to influence biodegradation capabilities and having an impact on their overall ecological competitiveness [28, 29], our objective in this study was to obtain a genome-wide comparative analysis of M213 such that a comprehensive understanding of the metabolic and evolutionary history of this strain can be ascertained. This study, encompassing intertwined approaches of genomics, metabolic and bioinformatic analyses, revealed several noteworthy features of strain M213, including a peculiar pathway for NAP biodegradation. Overall, the unique genomic organization and catabolic versatility of M213 likely facilitated its colonization and survival in the soil habitat from where it was isolated, which was co-contaminated with oil hydrocarbons and other toxic pollutants.

## Materials and Methods

### Whole Genome Sequencing, Assembly and Annotation

The whole genome sequence from strain M213 was obtained and assembled as described in our previous study [27]. Gene prediction and annotation was accomplished using the Integrated Microbial Genomes Expert Review (IMG/ER) [26]. Additionally, genome analysis was performed via RAST (http://rast.nmpdr.org/) and NCBI’s Prokaryotic Genome Automatic Annotation Pipeline (PGAAP) server (http://www.ncbi.nlm.nih.gov/genome/annotation_prok/). The functions of the predicted protein-coding genes were annotated using NCBIs non-redundant (NR) and Clusters of Orthologous Groups of proteins (COGs) databases, respectively.

Additionally, the draft genome sequence of strain M213 was reordered using the complete genome sequence of the type strains *R*. *opacus* B4 and *R*. *jostii* RHA1 as the reference genomes. For this purpose, r2cat (related reference contig arrangement tool) was used [30], which facilitated the comparative genomics of strain M213, especially at the syntenic level.

### Pulsed Field Gel Electrophoresis of Strain M213

To resolve the replicons in strain M213, the CHEF Genomic DNA Plug Kit was used according to manufacturer’s instructions (Biorad Life Science Research, CA) with the following modifications: *R*. *opacus* strain M213 was cultivated in LBP media (per L: 10 g peptone, 10 g NaCl, 5 g yeast extract) for 36 h; subsequently the culture was pelleted and concentrated from 250 ml into 50 ml fresh LBP media containing 180 μg/ml of chloramphenicol. Cells were further incubated for 1 h and then pelleted, and concentrated with the cell resuspension buffer to an OD of 20. Two ml of this concentrated cell suspension was added to 2 ml 2% clean cut agarose at 60°C for a final OD of 10 and added to gel plug molds. Gel plugs were added to lysozyme buffer with lysozyme (100 μg/ml) + 180 U of both lysostaphin and mutanolysin and incubated for 2 h at 37°C with shaking. Plugs were washed in sterile H_2_O and added to Proteinase K overnight at 50°C. Washes, including PMSF to inactivate Proteinase K were performed and plugs were stored in 0.5X TBE. BioRad CHEF PFGE was employed using the following parameters: 1% BioRad MegaBase agarose, 0.5X TBE buffer, 14°C, Switch Time 50–90, 5.5V for 22 h, 1 plug of Biorad PFGE lambda and/or *S*. *cerevisiae* ladder was added to the first well(s). The gel was stained in ethidium bromide for 1 h and rinsed with water before visualization and excision of the bands for qPCR analysis. Bands were gel excised from 3 replicate runs and DNA was extracted after bead-beating the slices at speeds 4, 5 and 6 for 1 min duration at each speeds. DNA extractions from each of triplicate gel slices was performed using the BioRad PFGE kit following the manufacturer’s protocol (BioRad Laboratories).

### Optical Mapping

Genomic DNA extracted from *R*. *opacus* M213 was loaded onto "MapCards" and input to the Argus optical mapping system (OpGen, Gaithersburg, Maryland). After restriction digestion with *Nco*I, high resolution imagery of the digested fragments were obtained. The individual fragments were assembled into a complete, or nearly-complete, single molecular restriction map. Multiple major maps were obtained for *R*. *opacus* M213, in part due to large extra-chromosomal elements (i.e. mega-plasmids). These optical maps were compared to *in silico* digests of *de novo* assembled genomic sequences generated through Illumina paired-end sequencing. Contigs with homology to known metabolic genes were identified in sequence-derived assemblies, and these fragments were categorized as either genomic DNA or plasmid-DNA based on optical mapping results.

### Bioinformatic Analyses

Resources utilized in this study for genome predictions and comparisons of strain M213 with other sequenced rhodococcii included those offered by NCBI (http://www.ncbi.nlm.nih.gov/) and Integrated Microbial Genomes Expert Review (https://img.jgi.doe.gov/cgi-bin/er/main.cgi). COGs from M213 and other rhodococii were compared using the Function Category Comparison tool using IMG/ER; each COG represents a protein that is an ortholog or direct evolutionary counterpart among genomes as they evolve over time. As with PFAM, IMG computes top COG hits using RPS-BLAST on PSSM's provided by CDD. Genes encoding phage integrases, transposases, and insertion sequence (IS) elements, transporters, transcriptional regulators, chaperones as well as selective biodegradative gene classes and other functional genes were identified manually using the annotated genome of strain M213 with the help of IMG-ER and NCBI portals. Additionally, gene sequences for the large subunits of naphthalene dioxygenase (narAa), phthalate 3,4 dioxygenase (phtAa) were mined from the whole genome sequence of strain M213 and phylogenetic trees were constructed using AromaDeg [31]. AromaDeg is a web-based repository of catabolic protein families such that when queried using a protein sequence of choice, AromaDeg builds a phylogenetic tree revealing clustering of the query sequence with a given catabolic protein family (http://aromadeg.siona.helmholtz-hzi.de).

Circular maps of the M213 genome and genomic islands (GEIs) were generated using the Web-based CGview program [32]. The average nucleotide identity (ANI) was calculated using the Web-based JSpecies program (http://imedea.uib-csic.es/jspecies/about.html). Clustered regularly interspaced short palindromic repeat (CRISPR) gene sequences were located in the genome of strain M213 from a publicly accessible CRISPRs database and software (http://crispr.u-psud.fr/Server/CRISPRfinder.php). The Clusters of Karlin signature skew, cumulative GC skew, and GC content were depicted using Artemis tools (sact_v9.0.5) [33]. Island Viewer was used to identify chromosomal deviations in GC content, known as genomic islands (GEIs) (http://www.pathogenomics.sfu.ca/islandviewer/) [34]. Additionally, the newly developed genomic island prediction software (GIPSy) [35] was utilized to evaluate the presence of different classes of GEIs in strain M213.

Venn diagrams, phylogenetic comparisons, core genome, pan genome, synteny plots and other comparative genomic features such as orthologous genes and distinction between core genes or singletons were analyzed using EDGAR (https://edgar.computational.bio.uni-giessen.de/cgi-bin/edgar_login.cgi?cookie_test=1&open=1) [36]. To further infer the evolutionary relatedness of M213 with close phylogenetic relatives, the genome of M213 was aligned with other rhodococii genomic sequences using Mauve (http://darlinglab.org/mauve/mauve.html) [37], which facilitates multiple genome alignments such that rearrangements and inversions from evolutionary events can be identified and comparatively visualized. Because genomic recombination events result in rearrangements, orthologous genomic regions of a bacterial strain may be reordered or inverted relative to another genome, which are clearly identified during Mauve analysis such that conserved genomic segments that appear to be internally free from rearrangements are shown as Locally Collinear Blocks (LCBs). Mauve was also used to produce dotplots showing chromosomal synteny between selected *Rhodococcus* strains.

In addition to the above approaches, Cloud Virtual Resource (CLoVR) was also utilized to run comparative genomics between M213 and other closely related *Rhodococcus* species. CLoVR- Virtual Machine (CLoVR-VM) module via the Data Intensive Academic Grid (DIAG) was used with the CloVR-Microbe pipeline (http://clovr.org/methods/clovr-microbe/), which facilitated gene finding, homology searches as well as automatic annotation (http://ae.igs.umaryland.edu/).

### Growth of Strain M213 on NAP and other Potential Metabolic Intermediates

M213 was grown on mineral salts media (MSM) consisting per liter of 4.8 g K_2_HPO_4_, 1.2 g KH_2_PO_4_, 1 g NH_4_NO_3_, 0.2 g MgSO_4_, 0.025 g CaCl_2_, 0.001 g Fe_2_(SO_4_)_3_ as shown before [25]. Briefly, a single colony of strain M213 was inoculated into 20 ml liquid LB broth and incubated at 30°C till an OD_600_ of 1.0 was obtained. The culture was then centrifuged for 10 min at 10,000 rpm, pellet rinsed three times with 10 ml of MSM and resuspended in 5 ml of MSM to obtain the concentrated cell suspension (CSS). A 1% stock of several potential metabolites in the NAP degradative pathway were prepared in methanol. To initiate the growth assay, CSS was diluted in MSM to a final OD of 0.15 and 270 μl of inoculum was added in each well of a Bioscreen honeycomb microtiter plate along with 30 μl of each substrate at 0.1% concentration. Assays were run in triplicate at 30°C in a Bioscreen C plate reader using the automatic stirring mode. To compare growth on different metabolites, the following controls were included with each run- cells without a carbon source and MSM without the cells, respectively. As a positive control, assays containing 0.1% glucose + cells were also run. Bioscreen C was set to measure optical density at 600 nm using the wide-band filter at every 6 hourly intervals over 6 days.

### Detection of Metabolic Intermediates during Growth of strain M213 on NAP

Concentrated cell suspension (CCS) of strain M213 was obtained as stated in the previous section and inoculated in 500 ml of MSM containing 0.05% NAP as the sole source of carbon and 50 ml fractions were collected at the beginning of the experiment followed by every day for a week. The metabolic intermediates from the supernatant collected at each time point was extracted using a separating funnel under neutral and acidic conditions with ethyl acetate as the solvent and samples were completely dried in a rotary evaporator (BÜCHI, Switzerland). Finally, the concentrated metabolic intermediates were resuspended in 200 μl of methanol and analyzed using a GC-MS.

GC-MS analysis was performed using the Agilent 7890B gas chromatograph equipped with an 5977A mass spectrometer and a J&W 2.5m particle trap column (27.5m x 0.53 mm, 20 μm thickness) (Agilent Inc., Santa Clara, CA). The injected volume of 1μl metabolic extract was used in a split less injection mode to maximize the amount of extracted metabolites in the column. Inlet and detector temperature was set at 250°C and 280°C, respectively. GC oven temperature began at 80°C and increased to 110°C at 8.0°C/ min, immediately followed by a ramp at 14.0°C/min to a final temperature of 310°C which was held for 3 min before run termination. Ultra-pure Helium was used as the carrier gas with flow rate of 0.5 mL/min. Metabolites of NAP biodegradation were identified based on comparison of the mass spectra with those of commercially available metabolic compounds and identification with Agilent’s mass spectral reference library (Mass Hunter AISt/NIMC Database—spectrum MS-NW-3989).

### Identification of the Naphthalene-dioxygenase (NDO) Gene in PFGE-resolved Replicons

Genomic DNA was extracted from strain M213 by growing it overnight in either LB or MSM supplemented with 0.1% NAP to induce the appropriate degradative genes. DNA extraction was performed using Power Soil DNA Isolation Kit (MoBio Inc.) and DNA concentration was estimated using the ND-1000 Nanodrop spectrophotometer (Starlab, USA). PCR-based identification of the NDO genes from the chromosome and plasmids- pNUO1 and pNUO2 were performed using BioRad iQ SYBR Green Mastermix and a Bio Rad C1000 thermocycler equipped with a CFX 96 real time system (Bio Rad Laboratories). PCR reactions were prepared using ready-to-go PCR beads (GE Healthcare, Fairfield, CT), with approximately 10 to 15 ng of template DNA, PCR grade sterile water and 0.4 pmol/μl of each primer sets that target either the narAa or narAb genes specific for α and β subunit of the naphthalene dioxygenase (NDO) catalytic component iron-sulfur protein (ISPNAR), which is the first enzymatic step of naphthalene biodegradation into 1,2-dihydroxynaphthalene. PCR with primer sets targeting narAa (1010F-5’-TACCTCGGCGACCTGAAGTTCTA-3’ and 5’-1611R-AGTTCTCGGCGTCGTCCTGTTCGAA-3’) or narAb (2031F-5’-GCACTCGTCACCGAGGATCTG-3’ and 2392R-5’-GATTGTTGTCTGATCTAGCAGCA-3’) resulted amplicon sizes of 625 and 404 bp, respectively [38]. The cycling conditions included an initial denaturing step at 95°C for 3 min, followed by 40 cycles of 94°C for 40 s, 55°C for 30 s, and 72°C for 60 s, as shown previously [39]. Amplicon size and purity were confirmed by gel electrophoresis.

### Gene Expression of Selected Genes of the NAP Biodegradative Pathway

A single colony of strain M213 was grown in 5 ml of NB supplemented with NAP for 24 h, followed by centrifugation, washing and resuspension of the resultant pellet in 5 ml of MSM. These cells were then inoculated into 500 ml of MSM medium containing 0.05% of either NAP or glucose (GLU). Fractions (50 ml) were collected similar to the GC-MS experiment- in increments of 24 hours over 6 days. Immediately after collecting the samples, they were centrifuged and the supernatants extracted for metabolic intermediates and bacterial pellets for RNA, such that formation of NAP degradative pathway metabolites can be correlated with the gene expression. Total RNA was extracted from microbial biomass using the Maxwell 16 LEV simplyRNA Blood kit (Promega, Madison, WI) according to the manufacturer’s instructions, and including an on-instrument DNAse treatment. Cells were pelleted and re-suspended in TE and lysozyme prior to running the extraction protocol. Total RNA was quantified using the Qubit RNA High Sensitivity Kit (Life Technologies, Grand Island, NY). Reverse transcription was performed using the High Capacity cDNA Reverse Transcription Kit (Life Technologies), employing random primers, according to the manufacturer’s instructions. Quantitative PCR was performed using custom designed hydrolysis probe assays synthesized by IDT (Integrated DNA Technologies, IA), and run using TaqMan Fast Advanced Master Mix (Life Technologies) according to manufacturer’s instructions on a Viia7 real-time PCR instrument.

Hydrolysis probe assays, shown in S1 Table, were obtained by querying the whole genome sequence of strain M213 (from IMG/ER) for the selected genes likely involved in the NAP degradative pathway and oligonucleotides were designed using the manufacturer’s recommendations (Integrated DNA Technologies, IA). After reverse transcription, genes for naphthalene dioxygenase large subunit (narAa), the Rieske iron-sulfur component of PAH dioxygenase, salicylate monooxygenase (SMO), phthalate 3,4 dioxygenase large subunit (phtAa), phthalate 3,4 dioxygenase small subunit (phtAb) and 16S rRNA were amplified separately in order to monitor the expression of these genes in both, NAP or GLU amended microcosms. The reported catabolic gene expression in NAP and GLU amended microcosms were obtained relative to the expression of a housekeeping gene (16S rRNA) along with running appropriate negative controls to eliminate the possibility of contamination.

### Nucleotide Sequence Accession Number

The whole genome sequence of strain M213 is available from DDBJ/EMBL/GenBank using the accession #AJYC00000000, version AJYC00000000.2.

## Results and Discussion

### Genomic and Catabolic Features of strain M213

**Fig 1:**
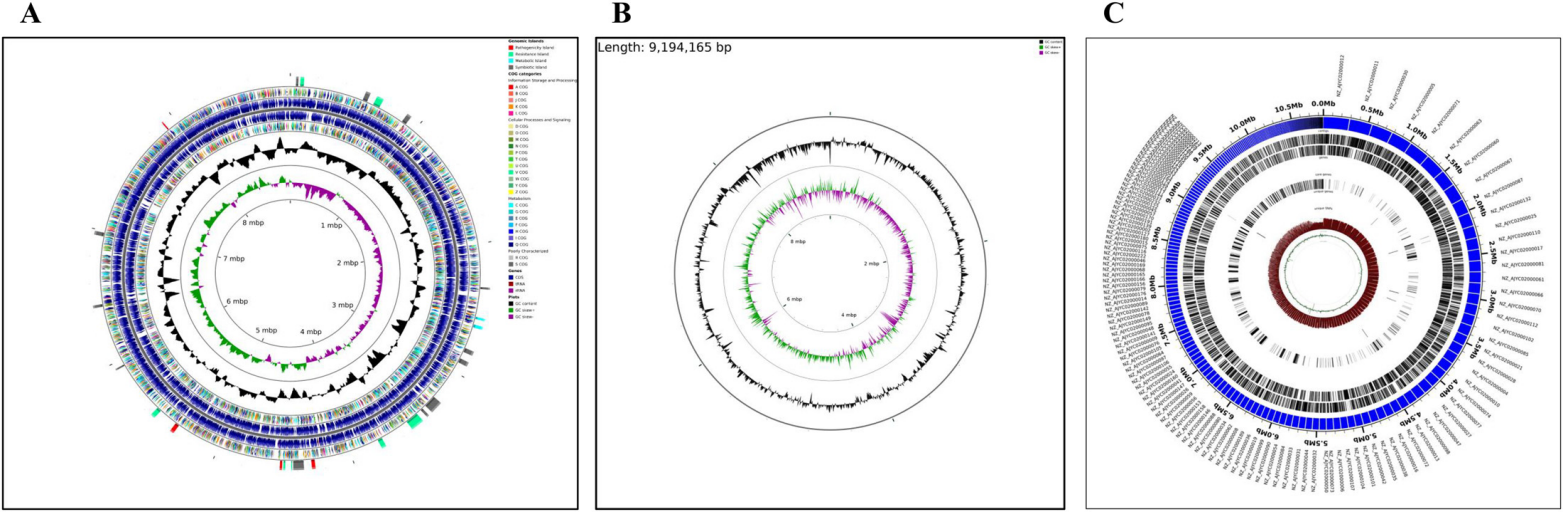
Genomic map of *R*. *opacus* strain M213. Shown are A, circular genome map of *Rhodococcus opacus* strain M213 with the first (outermost) and fourth rings depicting COG categories of protein coding genes on the forward and reverse strands, respectively. The second and third rings show the locations of protein coding, tRNA, and rRNA genes on the forward and reverse strands, respectively. The black plot depicts GC content with the peaks extending towards the outside of the circle representing GC content above the genome average, whereas those extending towards the center mark segments with GC content lower than the genome average. The innermost plot depicts GC skew. Both base composition plots were generated using a sliding window of 50,000 nt; B, genomic map of *R*. *opacus* strain M213 shown with the draft genome sequence re-ordered relative to *R*. *jostii* strain RHA1 as a reference organism; (C), A circleator-generated genomic map showing the total genes, single nucleotide polymorphisms (SNPs), along with the unique and core genome fragments of *R*. *opacus* strain M213 relative to other rhodococcii.

*Rhodococcus opacus* strain M213 was isolated from a fuel oil contaminated, North Idaho soil, using NAP as a sole source of carbon and energy [25]. We recently reported the whole genome sequence of M213, which is represented by approximately 87 M reads (~1000X average coverage), 483 contigs of 200 bases length or greater, an N50 of 79,111 bases, and an average coverage of >800X [27]. The genome size of M213 is typical of rhodococcii because these actinomycetes contain multiple replicons, many of which exist in a linear topology. The annotated genome of M213 was found to contain a total of 8680 putative coding genes with an approximate size of 9,194,165 bp, a G+C content of 66.72% and 72 RNA genes (8 rRNA, 48 tRNA and 16 other RNA genes) (Table 1). Key structural features of strain M213 such as the protein-coding sequences (CDSs), COGs, G-C content and GC skew are shown (Fig 1A). When contigs of strain M213 were re-ordered relative to the complete genome sequence of *R*. *opacus* B4 or *R*. *jostii* RHA1, a better assembly was obtained with the latter, (Fig 1B). In addition, the circleator feature embedded in CLOVR-Microbe was used to generate a graphical representation of the M213 genome (Fig 1C), which revealed an abundant set of unique genes and unique single nucleotide polymorphisms (SNPs), indicating the strong possibility of genome-wide differences of strain M213 with other actinomycetes.

**Table 1:** General features of the genomes of *Rhodococcus* species that were found to be functionally close to *R*. *opacus* strain M213 in this study.

Characteristics	*R*. *opacus* M213	*R*. *opacus* B4	*R*. *jostii* RHA1	*R*. *imetechnisis* RKJ300	*R*. *wratislaviensis* IFP2016
**NCBI No**	1129896	632772	101510	1165867	1195242
**Size (bp)**	9,193,504	8,834,939	9,702,737	8,231,340	9,687,645
**GC content (%)**	6158315 (66.99)	5974279 (67.62)	6499024 (66.98)	5533181 (67.22)	6486883 (66.96)
**Total no. of Genes**	8942	8259	9242	7733	9589
**Protein Coding Genes**	8630	8197	9156	7681	9527
**Proteins with Function Prediction**	6719	4861	5673	4847	7479
**DNA Coding Density (%)**	8135163 (88.49)	7979496 (90.32)	8703106 (89.70)	7003067 (85.08)	8213387 (84.78)
**Proteins assigned to COGs (%)**	6418 (71.77)	5761 (69.75)	6069 (65.67)	5611 (72.56)	5202 (54.25)
**DNA Scaffolds**	482	6	4	178	927
**Total RNA Genes**	50	62	86	52	62
**tRNA Genes**	48	49	52	50	53

The annotated genome of strain M213 by IMG/ER and RAST resulted in the assignment of approximately 75% of M213 genes to putative functional categories and the remaining genes as hypothetical proteins. Among the putative protein coding genes, 71.77% occurred as COGs, and 55% of these were present as paralog clusters. Furthermore, 22.5% and 20.7% of putative protein coding genes were connected to KEGG and MetaCyc pathways, respectively, indicating the presence of a cohort of metabolic genes in strain M213. COGs in M213 were further classified into 23 categories (S1 Fig) and 427 subsystems; the 5 most abundant subsystems related to transcription (n = 773); lipid transport and metabolism (n = 658); energy production and conversion (n = 642); amino acid transport and metabolism (n = 598); and secondary metabolites biosynthesis, transport and catabolism (n = 510). These findings suggest strain M213 to possess various genome-enabled processes related to energy production and secondary metabolite biosynthesis, respectively.

Growth of strain M213 on aromatic hydrocarbons and their metabolic intermediates, was assessed using both solidified [39] and liquid mineral salts media (S2 Fig), respectively. This revealed that M213 grew well on NAP as well as other hydrocarbons including phenol, toluene and chlorobenzoic compounds. Surprisingly, salicylate (SAL), the most commonly known metabolic intermediate in the NAP biodegradation pathway, did not support growth of M213. Conversely, vigorous growth was shown on other potential metabolites such as benzoate, gentisate, carboxybenzaldehyde, phthalate, hydroxyphthalate, and protocatechuate, respectively, (S2 Fig). Growth of M213 was also evaluated on variable concentrations of SAL- 0.01%, 0.1% and 0.5%, respectively, to ensure that 0.1% of salicylic acid was not toxic to M213 which might preclude growth. However, strain M213 was unable to support growth on SAL as the sole carbon source, even at variable concentrations [39].

### Replicons in Strain M213

Members of the genus *Rhodococcus* contain both, circular and linear plasmids, and in several cases, these replicons have been shown to possess catabolic functions [7, 8]. Pulsed field gel electrophoresis (PFGE) analysis showed three different replicons in strain M213—the chromosomal fraction, a 750Kb megaplasmid (pNUO1), and a smaller ~350Kb megaplasmid (pNUO2) (S3 Fig) [25].

**Fig 2:**
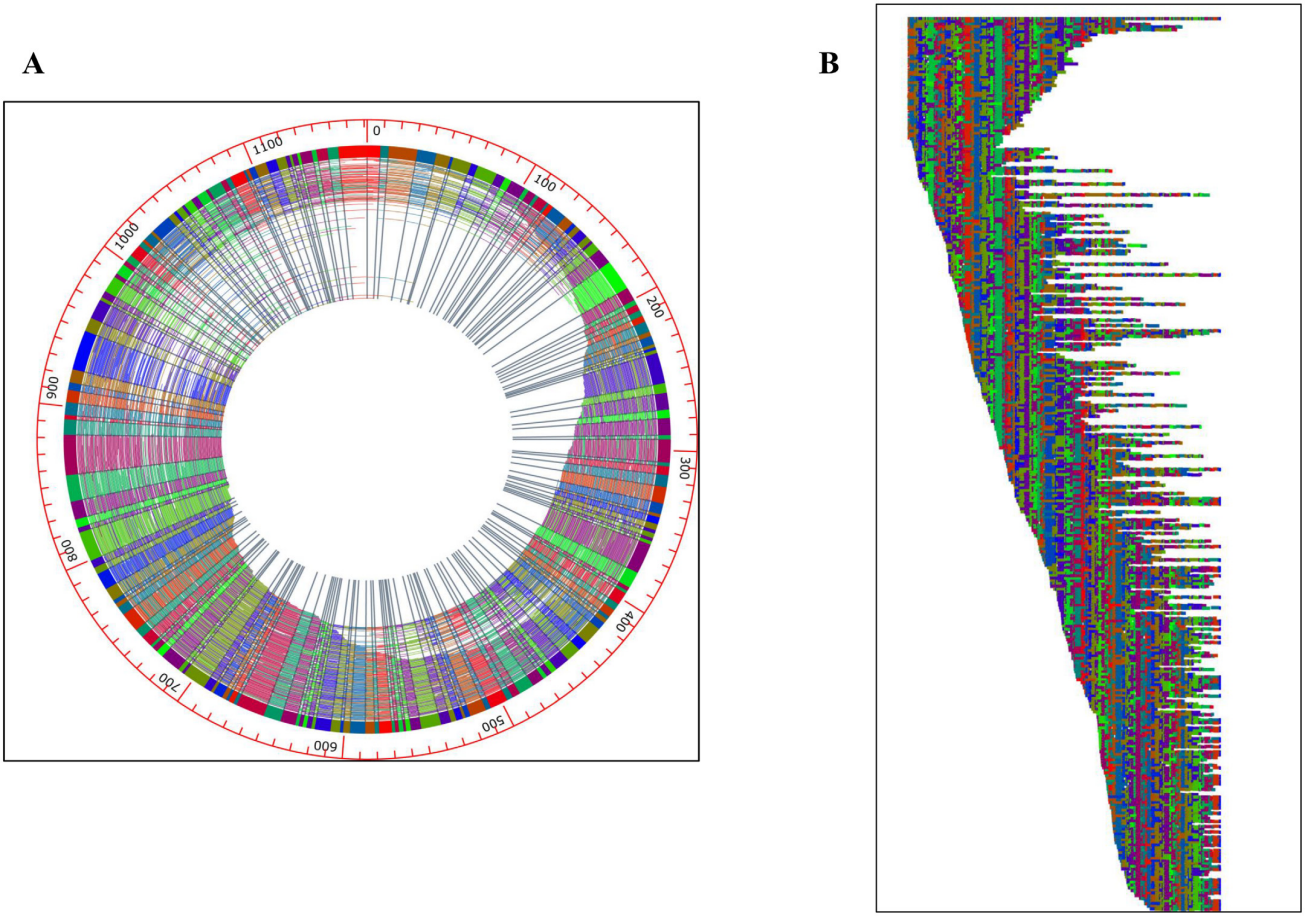
Optical maps of *R*. *opacus* strain M213 showing the presence of a circular megaplasmid (A) and a linear extrachromosomal element (B), respectively.

In addition to PFGE, whole genome mapping (optical mapping) was also performed to validate the primary *de novo* sequence assembly of M213 using an alternative, PCR and polymerase-independent technology, to improve the *de novo* assembly where possible and identify the location (chromosomal or plasmid-bound) of catabolic genes of interest. Optical mapping analysis revealed six distinct DNA fragments from strain M213, which included the presence of two megaplasmids- a circular, ~1.2 Mb plasmid, and a linear, ~0.73 Mb plasmid, along with a chromosome that was mapped into 4 pieces of 2.68, 2.21, 1.31 and 0.70 Mb (total optical map size ~7 Mb total) (Fig 2A and 2B). The total assembly of the optical map is approximately 8.8 Mb, leading to a small discrepancy in size between the *de novo* genome sequence analysis and optical mapping analysis. Unfortunately, even with the help of optical mapping, the large chromosome region of strain M213 could not be fully closed, which is likely due to the fairly large genomic size of strain M213. However, it is worth noting that optical mapping is more reliable in this situation because the plasmids are closed optical maps—which means that there is not much uncertainty in the final result. Sizing of megaplasmids from PFGE analysis can be unreliable because plasmids can remain supercoiled in the gel, and consequently, migrating as much smaller fragments.

### Genome-wide Comparative Analyses of Strain M213

**Fig 3:**
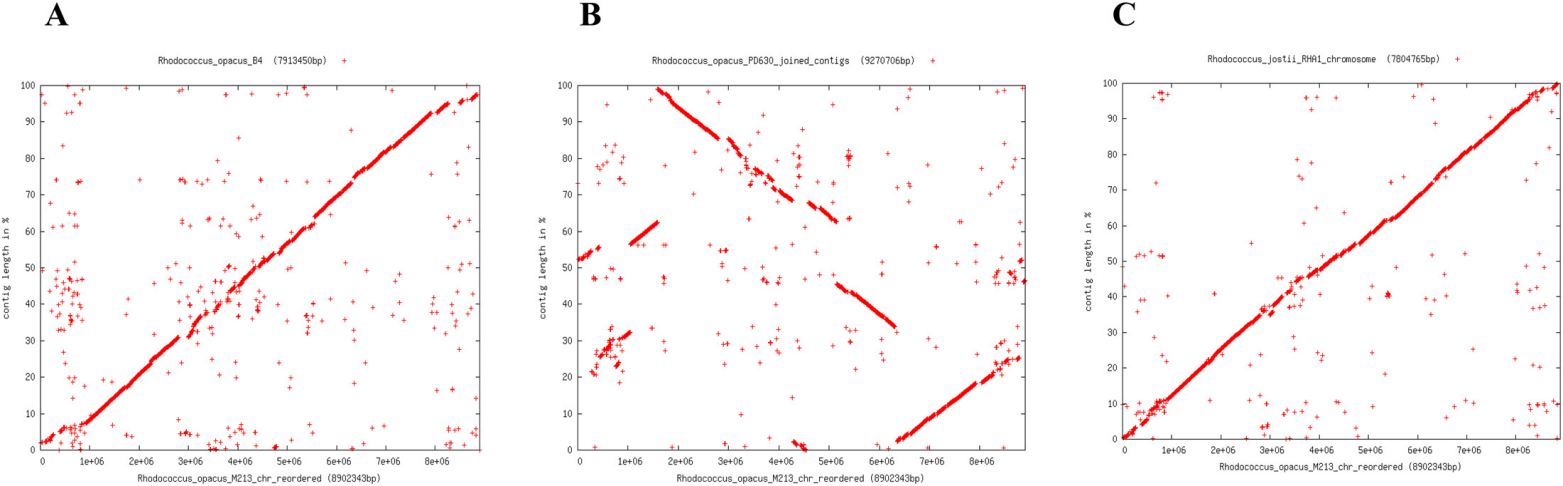
Whole genome-based synteny dot plots generated between *R*. *opacus* strain M213 and *R*. *opacus* strain B4 (A); strain M213 and *R*. *opacus* strain PD630 (B); strain M213 and *R*. *jostii* strain RHA1 chromosome (C), respectively.

A comparison of the M213 genome with other sequenced *Rhodococcus* species revealed a tight syntenic affiliation with the NAP biodegradative strain *R*. *opacus* B4 [17] (Fig 3A). Conversely, a significantly lower syntenic affiliation was observed for strain M213 with *R*. *opacus* strain PD630(Fig 3B). Syntenic regions are defined as homologous multi-gene regions in two or more genomes in which repertoire of genes are conserved, along with the potential conservation of transcription direction and linear gene order. Notably, when synteny was evaluated with the well-known biodegradative actinomycete- *R*. *jostii* [9], a greater syntenic affiliation of M213 was observed relative to strains B4 or PD630 (Fig 3C). Thus, this synteny-based evaluation strongly suggests that M213 consists of a mosaic of genes likely shared with other *Rhodococcus* species. In fact, synteny within closely related bacterial strains is a strong indicator of conserved gene function, hence, it is very likely that *R*. *opacus* strain M213 is functionally closer to the catabolically versatile *R*. *jostii* strain RHA1 relative to the *R*. *opacus* group consisting of strains B4 and PD630. Estimation of ANI values, which is a measure of genetic relatedness among bacterial strains, also revealed the closest similarity of strain M213 with *R*. *wratislaviensis* (98.39%) and *R*. *imtechensis* RKJ300 (98%) followed by *R*. *jostii* RHA1 (95%). Conversely, the least similarity was found with *R*. *opacus* B4 at 91.32%, respectively. Furthermore, when contigs from strain M213 were reordered relative to the genomes of *R*. *opacus* strain B4 and *R*. *jostii*, strain RHA1, M213 genome unexpectedly ordered more precisely with RHA1 than B4 (Fig 1B), another indication of closer affiliation with *R*. *jostii* and not *R*. *opacus* spp. The genome of *R*. *jostii* strain RHA1 has been completely sequenced and annotated [9], which revealed a genome of 9.7 Mbp with 9,221 predicted open reading frames (ORFs), which is one of the largest bacterial genomes sequenced to date.

**Fig 4:**
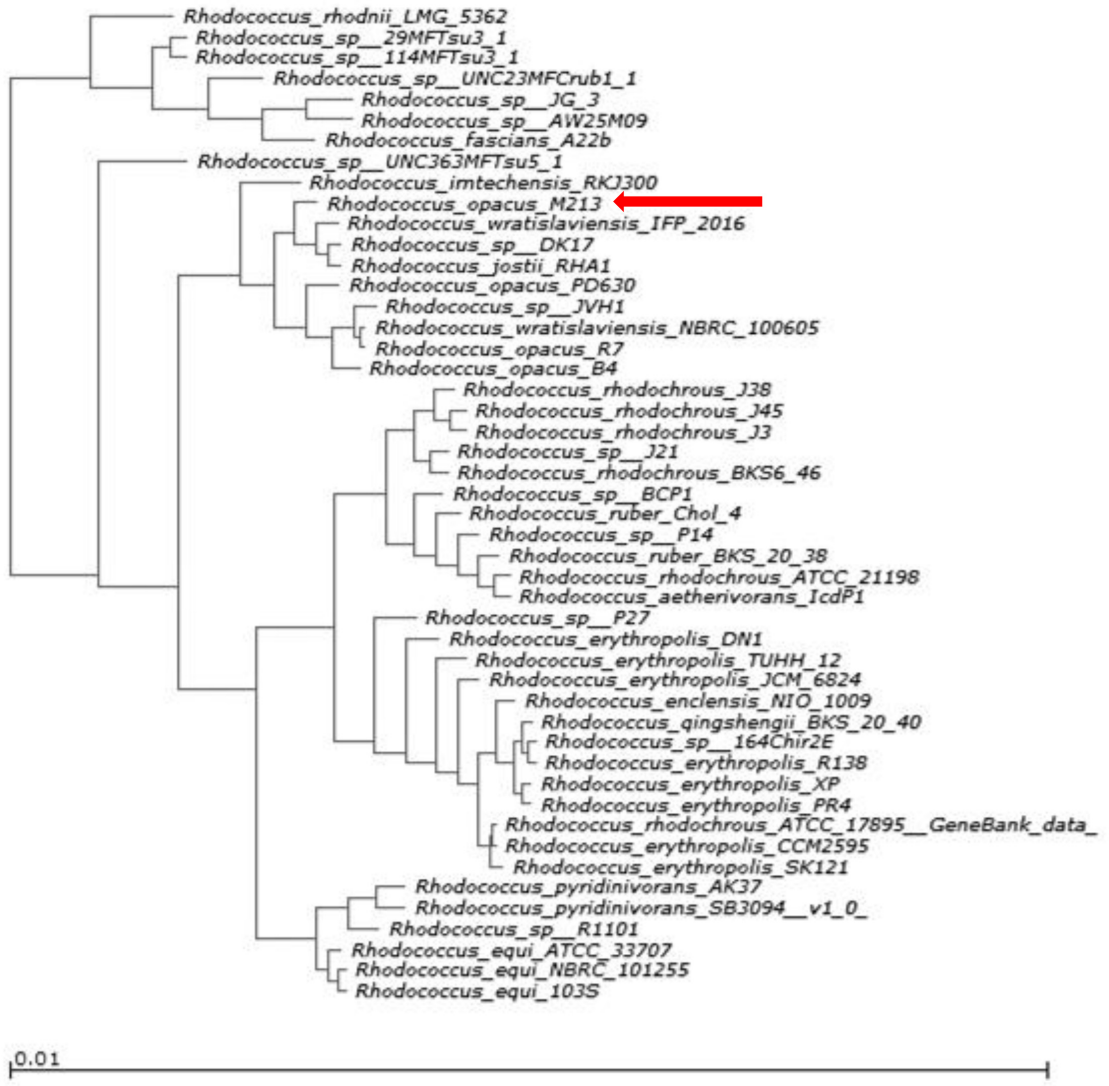
Phylogenetic tree obtained based on the presence of clusters of orthologous groups of proteins (COGs) from the genome of *R*. *opacus* strain M213 compared with a cohort of Rhodococcii for which whole genome sequences are available.

To better understand the functional affiliation of strain M213, COGs and KEGG pathway profiles of a cohort of 47 *Rhodococcus* species, for which either draft or complete genome sequences are available, were used to generate a hierarchical phylogram. Note that ortholog genes are believed to be the direct evolutionary equivalents between different bacteria that are related by vertical descent; this is in contrast to paralog genes, which appear within a genome and are related by duplication events. Thus, in general, orthologous proteins have similar domain architecture and function. COG-based hierarchical clustering showed M213 to align closer with *Rhodococcus wratislaviensis* strain IFP 2016, *R*. *imtechensis* RKJ300, *Rhodococcus* sp. DK17 and *R*. *jostii* RHA1 but not with *R*. *opacus* strains B4, R7 or PD630 (Fig 4), as also demonstrated by the previous synteny-based analysis (Fig 3). A similar result was obtained by analyzing M213 genome sequence via the CLoVR-Microbe comparative genomics capability (data not shown). Note that strain RHA1 was isolated from lindane-contaminated soil and is best known for its ability to degrade PCBs and an unusually broad range of organic compounds [9, 40, 41]. Similarly, both, strains IFP2016 [42] and DK17 [43] have broad catabolic potentials including genes for the bodegradation of benzene, toluene, ethylbenzene, m- and o-xylene, octane, hexadecane, cyclohexane, cyclohexanol and naphthalene. Strain RKJ300 can degrade several nitroaromatic compounds such as 4-nitrophenol, 2-chloro-4-nitrophenol, and 2, 4-dinitrophenol [44]. Based on COG analysis, it appears very likely that a mosaic of biodegradative genes have been recruited by strain M213, as reflected by its functional similarity with strains RHA1, IFP2016, DK17 and RKJ300, respectively.

Because the COG-based analysis showed M213 to be functionally closer to *R*. *jostii* strain RHA1, *R*. *wratslaviensis* strain IFP2016, and *R*. *imtechensis* strain RKJ300, further genomic comparisons of these specific rhodococcii were performed along with *R*. *opacus* B4 (the type strain of the opacus species); these comparisons are summarized in Table 1 and S2 Table. This comparative analysis showed that many features among these rhodococcii are quite similar; such as their genome sizes (between 9702737 bp to 8231340 bp), total numbers of gene (between 7,733 to9,589), number of protein coding genes (between 7,681 to 9,527), and total tRNA gene numbers (between 48 to 53). Information obtained from such analyses can provide clues on the evolutionary paths of these actinomycete species and further bolsters ourunderstanding on the recruitment mechanisms of biodegradative genes in Gram-positive bacteria, an area of research on which little information currently exists.

A detailed COG-based comparison revealed that all of these rhodococcii contain similar proportions of protein-coding genes (99%) that grouped into mostly 23 specific functions (S1A–S1C Fig). Specifically, when M213 was compared with *R*. *imtechensis* RKJ300, 7 COG categories falling under general function prediction, energy production and conversion, amino acid metabolism and transport, cell cycle control, cell motility, and RNA processing and modification were different by more than 15% (S1A Fig). Similarly, COGs in strain *R*. *wratislaviensis* IFP2016 that differed from M213 belonged to 13 different categories (S1B Fig). Surprisingly, only 4 COG categories (intracellular trafficking, secretion, and vesicular transport, cell motality, RNA processing and modification and chromatin structure and dynamics) were different between strains M213 and RHA1 (S1C Fig); similar to the previous synteny-based evaluation, this COG-based analysis reinforces strain M213’s tight functional affiliation with *R*. *jostii* RHA1 and not with its expected taxonomic relatives from the *opacus* species.

Using the Artemis Comparison Tool (ACT) available within the IMG/ER system, a pairwise genomic sequence comparison of strain M213 vs. *R*. *jostii* RHA1, *R*. *opacus* B4, *R*. *imtechensis* RKJ300, *R*. *wratslaviensis* IFP6 and *Rhodococcus* sp. DK17 analysis revealed the identity of a 7804765 bp genomic segment from strain M213 that was homologous to the chromosome of strain RHA1. This analysis also revealed close homology of a 1123075 bp genomic fraction from M213 with RHA1 plasmid pRHL1 along with a 442536 bp fraction with the RHA1 plasmid pRHL2 and 332361 bp fraction with the RHA1 plasmid pRHL3, respectively. Of major interest was a 286330 bp fragment containing 260 genes from *R*. *jostii* RHA1 genome, which mapped strongly with contig #12; the largest sized contig from the whole genome sequence of strain M213 (Fig 1C). When a dotplot of nucleotide based comparison using nucmer was generated (data not shown), this linear contig was confirmed to contain several biodegradative genes such as the NADH:flavin oxidoreductase (EC:1.5.1), aromatic ring hydroxylases (EC:1.14.14.9), dioxygenase and related ring-hydroxylating dioxygenases—additional evidence that several biodegradative genes are plasmid-encoded in M213. This bioinformatics-based evidence is in line with a previous study conducted on M213 that showed strong hybridization of cloned *edo*D with with pNUO1, the larger of the two plasmids present in M213, suggesting that genes involved in catabolic activity are carried by this very large plasmid in strain M213 [25].

The above stated COG-based analysis on strain M213 mirrors previous studies which show that the phylogeny of *Rhodococus* species may not adhere to those reflected alone by 16S rRNA based analysis [45, 46]. Specifically, in a survey conducted using several known genomic characteristics of *Rhodococcus* genus, it was demonstrated that an isolate of *R*. *opacus* clustered with *R*. *jostii* as opposed to seven others that clustered with *R*. *koreensis*, along with other two clustering with *R*. *wratislaviensis* [45], respectively. In fact, more recently, rhodococcal whole genome sequences were used by Creason et al. [46] to reconcile the dichotomy observed in the phylogeny of *Rhodococcus* genus, by using average nucleotide identity (ANI) and found that seven distinct *Rhodococcus* clusters were formed with inter-group comparisons that exceeded ANI values of 70–75%, a range that is typically found between members of the same genus [46]. Findings from this study suggested that the genus *Rhodococcus* can be represented by as many as 20 distinct species. In particular, Clade IV presented by the study of Creason et al. clearly show that it has two singletons and two subgroups; the first subgroup was represented by *Rhodococcus jostii* strain RHA1 which also included *Rhodococcus* spp. DK17 and JVH1 [46]. The second subgroup consisted of *Rhodococcus opacus strains* M213 and PD630 along with the type strain of *Rhodococcus imtechensis* RKJ300 and *Rhodococcus wratislaviensis* IFP 2016. The other singleton isolates were *R*. *opacus* B4 and *R*. *wratislaviensis* NBRC 100605. Thus, our whole genome sequence based analysis of strain M213 is directly in line with those presented by Creason et al. [46], demonstrating that strain M213 affiliated more closely with *Rhodococcus imtechensis* RKJ300 and *Rhodococcus wratislaviensis* IFP 2016 but not with *R*. *opacus* strains such as B4, as would be expected.

**Fig 5:**
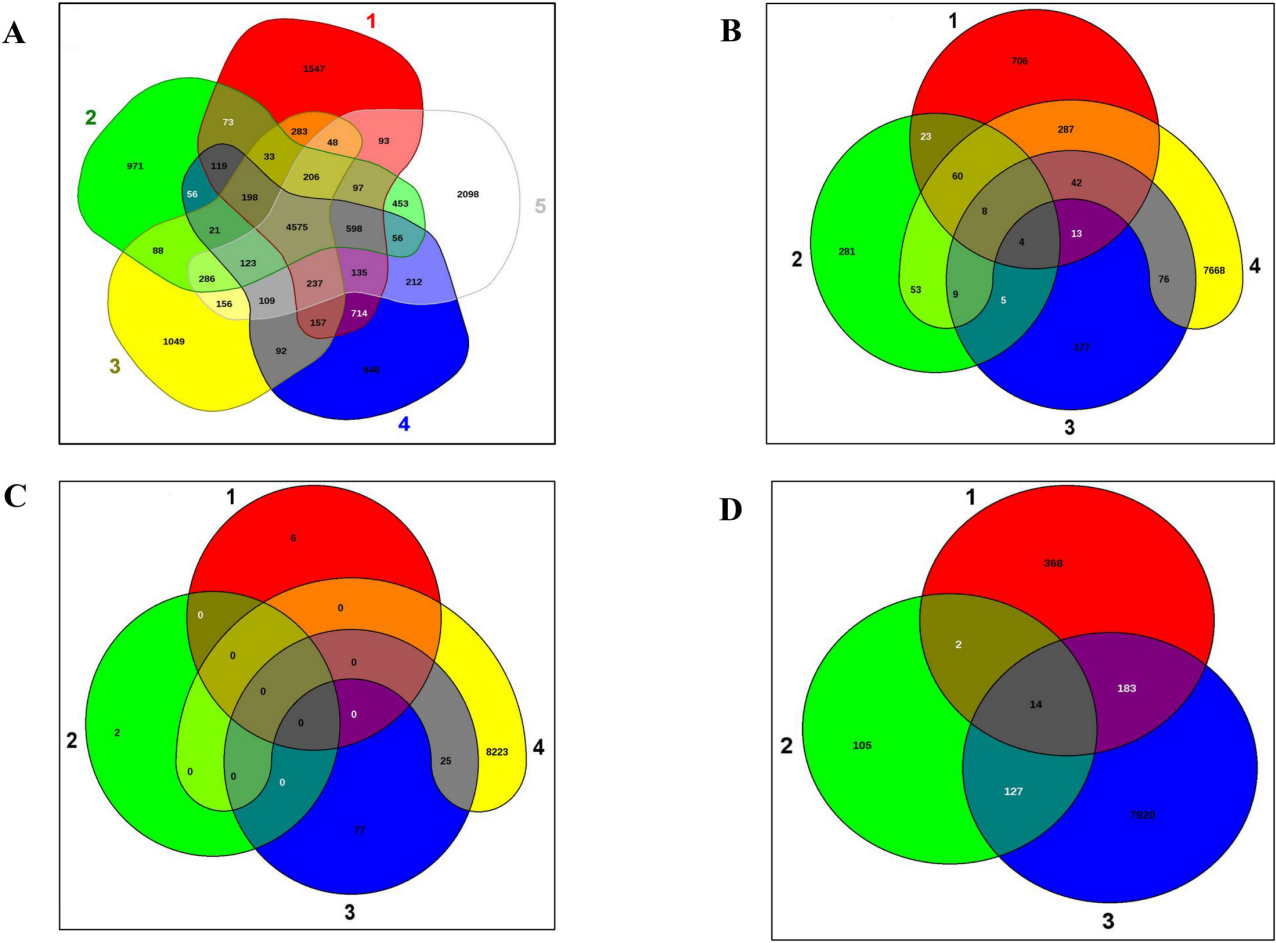
Whole genome based Venn diagrams generated between *R*. *opacus* strain M213 with its closest functional relatives. A, Venn diagram sectors belong to 1, *Rhodococcus jostii* strain RHA1; 2, *Rhodococcus opacus* strain M213; 3, *Rhodococcus imtechensis* RKJ300; 4, *Rhodococcus wratislaviensis* strain IFP2016 and 5, *Rhodococcus* sp. strain DK17. The number of singleton genes appear in red, green, yellow, blue and white areas for strains 1–5 listed above -along with their core genomes (centered gray area); B, Venn diagram of plasmids from *Rhodococcus jostii* strain RHA1; shown are pRHL1 (sector 1), pRHL2 (sector 2) and pRHL3 (sector 3), and genome of M213 (sector 4); C, Venn diagram of plasmids from *Rhodococcus opacus* strain B4; shown are pKNR1 (sector 1), pKNR2 (sector 2), pKNR (sector 3) and genome of M213 (sector 4); D, Venn diagram of plasmids from *Rhodococcus opacus* strain B4; shown are pROB1 (sector 1), pROB2 (sector 2), and genome of M213 (sector 3), respectively.

Additionally, EDGAR based genome-wide comparisons between *R*. *opacus* strain M213, *R*. *wratslaviensis* strain IFP2016, *R*. *imtechensis* RKJ300, *R*. *jostii* RHA1 and *Rhodococcus* sp. DK17 revealed that M213 harbored 971 distinct genes not present in any of these other rhodococii (Fig 5A). This analysis facilitated the detection of 4575 CDSs shared by the five isolates and a number of distinctive genes for each species, when compared to each other: RHA1 (1547 CDSs); RKJ300 (1049 CDSs); IFP2016 (948 CDSs); and *Rhodococcus* sp. DK17 (2098 CDSs), respectively. The M213-encoded distinct genes constituted approximately 11% of its total genome size with much of this metabolome found to encode degradative enzymes including several dioxygenases, decarboxylases, hydroxylase, and dehydrogenase. Moreover, two putative plasmid partitioning proteins were also found to be present unique to strain M213 confirming the presence of plasmid(s) in this strain, as was shown earlier using PFGE analysis (S3 Fig), respectively. When the pool of orthologous genes (4575 CDSs) shared by the five isolates were further surveyed, we found approximately 90 shared oxygenase coding genes and several other catabolic genes, which confirms the strong catabolic potential that is possessed by soil actinomycetes, especially these groups of *Rhodococcus* strains.

Because biodegradative traits are often encoded on plasmids, comparisons were also drawn between the genome of M213 and plasmid sequences of strains RHA1 and B4 (Fig 5B and 5C). This revealed the presence of 287 orthologous genes between strain M213 and RHA1-encoded plasmid- pRHL1 (Fig 5B); some of these genes have been previously linked with biodegradative functions such as catechol 2,3-dioxygenase, benzene dioxygenase ferredoxin reductase subunit, 2-nitropropane dioxygenase, dioxygenase Rieske iron-sulfur component, and extradiol dioxygenase, type I. We also found several transposases, including IS4 and the putative transposase y4qE common between these strains (data not shown). When comparison of M213 to plasmids in *R*. *opacus* B4 was performed, we found only 25 genes common with plasmid pKNR1 (Fig 5C); conversely, 183 genes were similar to those previously mapped on plasmid pROB01 in strain B4 and 127 genes were common with the second plasmid- pROB02 in strain B4 (Fig 5D); some of these genes included alkane 1-monooxygenase, alkene monooxygenase rubredoxin, and alkene monooxygenase rubredoxin reductase.

Other orthologous genes included several transposases such as IS3 family transposase, putative transposase orfB for an IS element, putative transposase for an IS element and a hypothetical transposase protein paralog, suggesting active role played by these mobile genetic elements in genomic rearrangements of strain M213. Moreover, we found approximately 19 transposases common between strains M213 and B4, including putative transposase orfB for IS element, IS3 family transposase, putative transposase/integrase as well as an identical paralog for transposase (data not shown). Such transposases and integrases mediate transposition events i.e. the recombination events in which disparate DNA segments move between non-homologous sites within the genome of an organism. This is strong evidence that transposases, integrases and recombinases likely facilitated genomic reshuffling of strain M213, causing it to deviate from its expected taxonomic position with the other *R*. *opacus* species.

### Genomic Reshuffling and Rearrangements Identified in M213

One characteristic feature commonly observed within most bacterial genomes is the presence of highly conserved, direct DNA repeat arrays called “clustered regularly interspaced short palindromic repeats,” (CRISPRs) along with interspersed similar-sized spacers. CRISPRs along with the CRISPR-associated (cas) proteins [47] protect against bacteriophage predation or plasmid incorporation through an RNA-mediated mechanism that is encoded by the spacers. Moreover, the presence of CRISPRs generally serve as an indicator of previous bacteriophage encounters because new spacers become integrated at the 5′ end of the array consequent to the acquisition of foreign DNA element preventing further bacteriophage attacks [48]. When the genome of strain M213 was surveyed for the presence of CRISPRs, 1 confirmed and 14 potential CRISPRs were found (data not shown). Conversely, when the genomes of several other sequenced rhodococcii were surveyed for CRISPRs, none were found to contain any confirmed CRISPRs. When flanking sequences from the confirmed CRISPER in M213 was studied using BLAST, a match with *R*. *opacus* R7 was found, indicating potential recruitment of the CRISPR from strain R7 which was isolated from a PAH contaminated soil [18].

**Fig 6:**
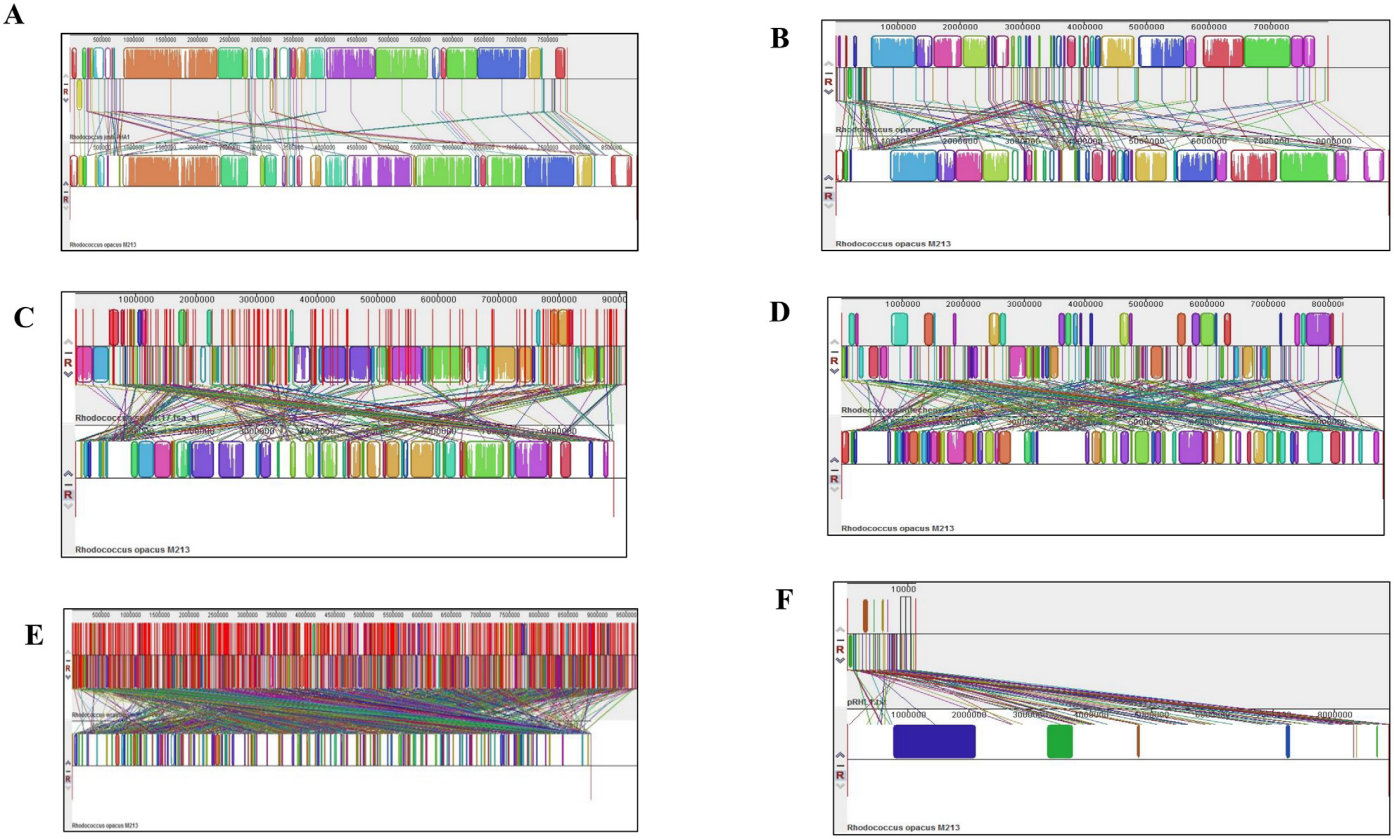
Whole genome comparative alignment of *R*. *opacus* strain M213 with *Rhodococcus jostii* strain RHA1. (A); *Rhodococcus opacus* strain B4 (B); *Rhodococcus* strain DK17 (C); *Rhodococcus imtechensis* RKJ300 (D); and *Rhodococcus wratislaviensis* strain IFP2016 (E), respectively. Also shown is the whole genome comparative analysis of *R*. *opacus* strain M213 with plasmid pRHL1 from *Rhodococcus jostii* strain RHA1 (F). Each of the genome sequence analyzed is presented horizontally with the scale showing the sequence coordinates and the conserved segments represented as the colored blocks which are connected across genomes. Blocks that are shifted downward in a genome represent those segments that are inverted relative to the reference genomes. The aligned region is in the forward orientation relative to the first genome sequence if a block lies above the center line; blocks below the center line indicate regions that align in the reverse complement (inverse) orientation of the reference genome. Genomic regions falling outside the blocks lack a detectable homology among the genomes analyzed. Within each block is shown the similarity profile of the genome sequence such that the height of the similarity profile corresponds to the level of conservation in that specific region of the genome. White areas represent those genomic regions that did not align well between the input genomes and likely contain sequence elements specific to a particular genome.

The genome sequence of M213 (re-ordered relative to *R*. *jostii* strain RHA1) was also compared individually with *R*. *wratislaviensis* IFP6, *R*. *jostii* RHA1, *R*. *opacus* B4, *R*. *imtechensis* RKJ300, *R*. *wratslaviensis* IFP6 and *Rhodococcus* sp. DK17 using Mauve (Fig 6A–6E). Mauve is a system for efficiently constructing multiple genome alignments in the presence of large-scale evolutionary events such as rearrangement and inversion [37], which provides a basis for comparative genomics and evolutionary dynamics. Mauve analyses showed the presence of several crisscrossing locally collinear blocks (LCBs), suggesting the complicated rearrangement profiles of M213 relative to related rhodococcii genomes. Regions that are inverted relative to *R*. *opacus* M213 appear shifted below a genome's center axis (Fig 6).

Visual inspection of the rearrangement pattern for M213 relative to other rhodococcii revealed several instances of local overlapping inversions characteristic of symmetric inversion about the terminus, distinguishable as a “fan” pattern of crossing lines. In fact, 86 LCBs with a minimum weight of 74 were found between M213 and *R*. *jostii* RHA1 (Fig 6A), 133 LCBs with a minimum weight of 404 with *R*. *opacus* B4 (Fig 6B), 238 LCBs with a minimum weight of 50 with *Rhodococcus* sp. DK17 (Fig 6C); 274 LCBs with a minimum weight of 128 with *R*. *imtechensis* RKJ300; (Fig 6D), 965 LCBs with a minimum weight of 48 with *R*. *wratislaviensis* IFP2016 (Fig 6E), respectively. This reiterates our earlier stated findings to demonstrate that strain M213 is closest to *R*. *jostii* strain RHA1 in terms of homologous regions that do not contain any rearrangements i.e., LCBs.

In addition, Mauve comparisons between M213 and plasmids from rhodococcii were also made, as shown in Fig 6F. Because the presence of 287 orthologous genes between M213 and RHA1-encoded plasmid- pRHL1 was previously shown by EDGAR analysis (Fig 5B); Mauve analysis between the genome of strain M213 and *R*. *jostii* encoded pRHL1 plasmid identified 78 LCBs with a minimum weight of 111 (Fig 6F), further providing rigorous proof that M213 harbors plasmid-encoded genes and many of these are likely similar to those found on pRHL1 with metabolic activities against a broad-range of hydrocarbons.

### Genome Islands (GEIs) Identified in M213

It is well recognized that bacterial genomes consist of a core set of genes that encode for essential metabolic functions, along with a plethora of acquired genes from the bacterium’s evolutionary lineage by horizontal gene transfer (HGT), providing the host with evolutionary adaptive traits and genome plasticity. Many such HGT-acquired accessory genes occur as orthologous blocks referred to as genomic islands (GEIs) [29]. GEIs have been mainly attributed to render virulence or antibiotic resistance to the host bacteria, but the recent body of genome sequencing studies offer other adaptive functional traits brought about by GEIs to the host bacterium. Thus, GEIs are now broadly classified into 4 categories based on their associated functions- pathogenicity islands (PAIs), harboring virulence genes [49]; metabolic islands (MIs), genes for the biosynthesis of secondary metabolites [50]; resistance islands (RIs), genes that code for resistance, for example, against antibiotics [51]; and symbiotic islands (SIs), those genes that facilitate symbiotic associations of the host with other micro- and macroorganisms [52].

Specific to biodegradation, a number of GEIs have been correlated with the biodegradation of chlorinated and nitroaromatic compounds. For example, a 100 kb *clc* GEI was found to degrade chlorocatechols and aminophenols [53], a 90 kb *bph-sal* element [54] and a 55 kb biphenyl catabolic transposon Tn4371 [55] degraded biphenyl, a 100 kb Tn3-like SN2 transposon degraded naphthalene [56], and an unclassified GEI associated with naphthalene degradation in *Polaramonas napthalenivorans* strain CJ2 [57]. More recently, the entire genes for the phenanthrene degradative pathway were mapped on a novel ~232 kb GEI, called the phn island [58].

Further analysis of the genome of M213 led to the identification of as many as 154 total genomic islands, with traits of virulence, metabolism, resistance and symbiosis, respectively (Table 2 and Fig 1A). These GEIs represent gene clusters acquired by M213, likely via relatively recent lateral gene transfer, as is suggested in bacterial evolution and adaptive mechanisms for survival [59]. It is interesting to note that many of these potential GEIs contain genes related to biodegradation of pollutants, including several monooxygenases and dioxygenases. We speculate that several of the above GEIs were likely acquired by M213 from other soil bacteria possessing versatile biodegradative abilities because a majority of GEI associated proteins showed homologues to other soil-dwelling bacterial genomes such as *Rhodococcus*, *Gordonia*, *Micromonospora*, *Pseudonocardia* etc.

**Table 2:** Characteristics of some of the key genomic islands (GEIs) identified from the whole genome sequence of *R*. *opacus* strain M213.

Locus_ID/Gene Name	Prediction Method	Predicted Function(s)
**GEIs Related to Biodegradation:**		
WSS_A02450 WSS_A02455 WSS_A02465	IslandPick	Naphthalene dioxygenase small and large subunits, cis-naphthalene dihydrodiol dehydrogenase
WSS_A02435	IslandPick	Putative naphthalene degradation regulatory protein
WSS_A07969	IslandPick	Phthalate 4,5-dioxygenase
WSS_A12338	IslandPick	3,4-dihydroxyphthalate decarboxylase
WSS_A12343	IslandPick	Phthalate dioxygenase ferredoxin reductase subunit
WSS_A12348	IslandPick	Phthalate 3,4-dioxygenase ferredoxin subunit
WSS_A12368	IslandPick	Phthalate 3,4-dioxygenase alpha subunit
WSS_A12353	IslandPick	2,3-dihydroxy-2,3-dihydrophenylpropionate dehydrogenase
WSS_A12378	IslandPick	Phthalate ester hydrolase (isochorismatase hydrolase)
WSS_A02315	IslandPick IslandPath-DIMOB	Homogentisate 1,2-dioxygenase
WSS_A12408	IslandPick	Terephthalate 1,2-dioxygenase alpha subunit
WSS_A12413	IslandPick	Aromatic ring dioxygenase beta subunit
WSS_A33715	IslandPick	FAD-binding monooxygenase
WSS_A12408	IslandPick	Terephthalate 1,2-dioxygenase alpha subunit
WSS_A12413	IslandPick	Aromatic ring dioxygenase beta subunit
WSS_A33715	IslandPick	FAD-binding monooxygenase
WSS_A02340	IslandPick	Cytochrome P450 monooxygenase
WP_037205976	IslandPick	Cyclohexanone monooxygenase
WP_005257442	IslandPick	NADH dehydrogenase
WP_005263192	IslandPick	2-nitropropane dioxygenase
WP_005258011	IslandPick	2-hydroxy-3-oxopropionate reductase
WP_005257988	IslandPick	Oxido-reductase
WSS_A20339 WSS_A27010	IslandPick	Cytochrome P450
WSS_A43735	IslandPath-DIMOB	Cytochrome P450
WSS_A02340	IslandPick/ IslandPath-DIMOB	Cytochrome P450 monooxygenase
WSS_A07974	IslandPick	Cytochrome P450 family protein
WSS_A25125	IslandPick	Cytochrome P450 CYP258
WSS_A11738	IslandPick/ IslandPath-DIMOB	Methylmalonate-semialdehyde dehydrogenase
WSS_A17476	IslandPick	Carboxymuconolactone decarboxylase
WSS_A02370 WSS_A07264	IslandPick	Catechol 2,3-dioxygenase
WSS_A12363	IslandPick	3-phenylpropionate dioxygenase subunit beta/ cinnamic acid ferredoxin subunit dioxygenase
WSS_A20329	IslandPick	FAD-dependent oxidoreductase
WSS_A30014	IslandPick	Monooxygenase FAD-binding protein
WSS_A30159	IslandPick	Nitric oxide dioxygenase
WSS_A37951	IslandPick	Hydroxyquinol 1,2-dioxygenase
WSS_RS27225	IslandPick	Nitrate reductase Z subunit alpha
WSS_RS29470	IslandPick	Nitrate reductase Z subunit beta
WSS_A14784 WSS_A17476	IslandPick	Carboxymuconolactone decarboxylase
WSS_A12428	IslandPick	4-hydroxybenzoate transporter
**GEIs Related to Horizontal Gene Transfer:**		
WSS_RS12770	SIGI-HMM	Transposase
WSS_A22098	IslandPick/ SIGI-HMM/ IslandPath-DIMOB	Putative plasmid partitioning protein
WSS_A22098	IslandPick/ SIGI-HMM/ IslandPath-DIMOB	Protein ParA
WSS_A43785	IslandPick	Transposase families IS111A/IS1328/IS1533
WSS_A16321 WSS_A25885 WSS_A29589 WSS_A09047	IslandPick	Phage integrase family protein
WSS_A29209 WSS_A29214	IslandPick/ IslandPath-DIMOB	Phage integrase family protein
WSS_A32440	IslandPick/ IslandPath-DIMOB	ParB family plasmid partitioning protein

The presence of high numbers of GEIs in M213 also provides clues about the genomic plasticity of this soil isolate, likely conferred by mobile elements such as integrases or transposases). Specifically, we identified at least 11 GEIs (Table 2) that encode genes associated with integrases, transposases, or phage infections (restriction modification systems); thus, these GEIs likely contributed to the overall pool of transposase and IS elements impacting genetic rearrangement and horizontal gene transfer in strain M213. In addition, NCBI annotated genome of strain M213 also revealed the presence of several phage-related proteins. Consistent with this, we found several GEIs to contain footprints of phages or their remnants including, but not limited to, phage integrase family protein, integrase core domain protein, and integrase catalytic subunit (Table 2). Moreover, using PHAST (Phage Search Tool), we found one intact (100% score) prophage region of 42.5 Kb in size within the genome of M213 containing 57 coding sequence (CDS) and a GC content of 65.47% (S4 Fig). The closest relative of this region is with the Giles Mycophage, which infects *Mycobacteria*, followed by the *Rhodococcus* phage REQ2, Fishburne phage for *Mycobacteria* and *Rhodococcus* phage REQ3. This analysis strongly suggests the likelihood of *R*. *opacus* M213 being attacked by several different groups of bacteriophages during its recent evolutionary trajectory, which further explains the presence of abundant mobile elements such as GEIs and CRISPRS within the genome of this soil-dwelling isolate.

It was also found that the core gene pool, which indicates evidence for recombination after a phage attack, were overrepresented in M213, as shown under the COG “L”- category (data not shown). This revealed that M213 contained relatively higher gene numbers (273) related to replication, recombination and repair (COG L), relative to other Rhodococcii strains. Thus, the presence of a higher number of GEIs in combination with CRISPRs, indicates that strain M213’s genome has likely undergone widespread genetic changes during its recent adaptive evolutionary history via bacteriophage attacks, horizontal gene transfer (HGT) mechanisms and genetic reshuffling. The complex patterns shown by the synteny dot-blot analysis of the genome of M213 (Fig 3) also indicates extensive genomic translocation, inversion, and insertion likely mediated by mobile genetic elements such as transposases, IS elements, phage integrases. This was further confirmed by performing a genome-wide comparison of M213 with several other sequenced *rhodococcii* using the Mauve program, especially in the form of reciprocal inversions. Furthermore, deviations from the mean values of cumulative GC skew and GC content in the genomic DNA of *R*. *opacus* strain M213 (Fig 1) provide additional evidence for structural rearrangements undergone by strain M213, in a fuel-oil contaminated soil habitat, from where it was isolated.

Of major note is the identification of several GEI-encoded genes in M213 that likely facilitates degradation of naphthalene (NAP) via a peculiar pathway (Table 2). Towards this end, our previous findings indicate that strain M213 does not metabolize NAP solely via salicylate but also through the formation of *o*-phthalate as a metabolic intermediate [27, 60], representing a significant divergence from previously described pathways for NAP metabolism, especially in mesophilic bacteria. The presence of some of the genes associated with this NAP biodegradative pathway were also found on GEIs in strain M213, such as the small and large subunits of naphthalene dioxygenase (NDO), the cis-naphthalene dihydrodiol dehydrogenase gene, and a putative naphthalene degradation regulatory protein (Table 2). This finding that GEIs harbor catabolic genes in strain M213 are in direct line with findings from *Pseudomonas putida* strain CSV86 where the upper pathway genes of NAP degradation were found to be part of a genomic island [61, 62]. Note that NDO is a well-studied multicomponent enzyme system that oxidizes naphthalene to cis-(1R, 2S)-dihydroxy-1,2-dihydronaphthalene but significant differences have been observed between NDO of Pseudomonads and Rhodococcii [63]. In addition to NAP, we also identified GEIs with genes for the degradation of phthalate, catechol, cinnamic acid ferredoxin subunit dioxygenase, 4-hydroxybenzoate, and several oxygenases, dioxygenases and cytochrome P450 related genes with the potential for biodegradative functions (Table 2). Furthermore, some of these biodegradative genes were found duplicated onto multiple GEIs in strain M213 (data not shown).

These bioinformatic lines of evidence for a peculiar pathway for NAP biodegradation in M213 are further supported by metabolic analyses i.e., GC-MS based measurements of metabolic intermediates resulting from the growth of M213 on NAP, which included both salicylate (SAL) and *o*-phthalate (OPA) (Table 3) and a suite of several other potential metabolic intermediates.

**Table 3:** Metabolic intermediates detected by GCMS over a 7-day growth period of *R*. *opacus* strain M213 when grown on 0.5% Naphthalene as the sole source of carbon and energy.

Retention Time (min)	GCMS {m/z (% relative intensity) [molecular ion]}	Identified Compound
4.77	122*(100) [M+], 121(93), 65(41), 39(41), 93(24), 76(24)	Salicylaldehyde
5.74	128*(100)[MH+],102(85),51(54),75(36)	Naphthalene
6.28	92*(100) [M+], 120(89), 138(46), 64(25), 63(20), 39(13), 65(13)	Salicylic Acid
6.50	104*(100) [M+], 76(85), 18(62), 50(43), 148(37), 38(19), 74(16), 17(13), 37(10), 75(10)	Phthalic anhydride
6.89	148*(100)[M+],120 (88),91(74),78(45)	Hydrocoumarin
7.203	118*(100) [M+], 146(83), 89(37), 90(37), 63(25), 62(10), 51(9.4)	Cinnamic Acid
7.207	118*(100) [M+], 146(80), 90(30), 39(25), 164(10)	2-hydroxy Cinnamic Acid
7.21	118*(100)[M+],146(69), 90(41), 89(40), 63(28), 62(11)	Coumarin
7.22	104*(100) [M+], 76(70), 163(55), 50(40), 148(20)	Phthalic Acid
7.937	107*(100) [M+], 149 (80), 121 (40), 208 (20), 134 (20)	2,3-dihydro 1,4-benzodioxin-6-Propanoate
8.52	131*(100) [M+], 176 (50), 77 (20), 103 (20)	3-methyl-2-benzofuran Carboxylic Acid
8.99	146*(100) [M+], 133(709), 105(520), 77(389), 51(202)	2-Carboxy Cinnamic Acid

**Fig 7:**
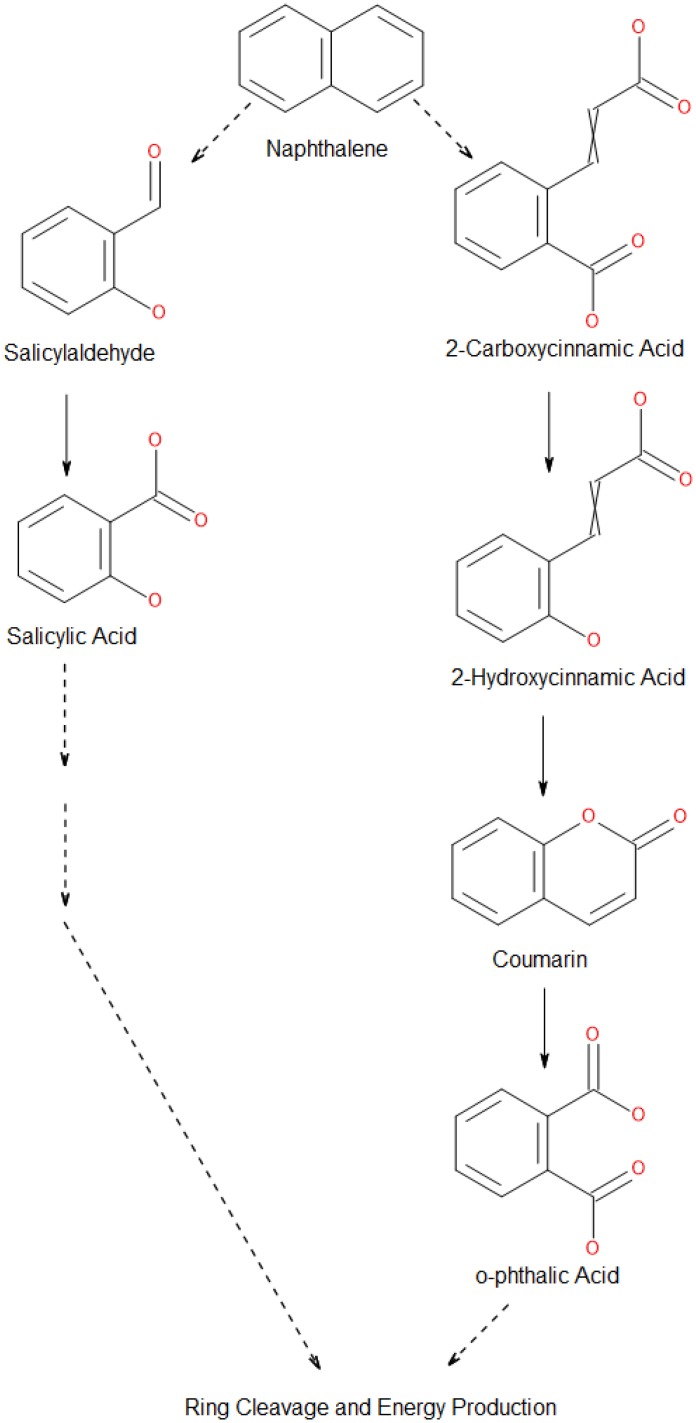
Shown is the peculiar biochemical pathway identified from *R*. *opacus* strain M213 utilized for the biodegradation of naphthalene (NAP), as revealed by an intertwined biochemical, genomics and bioinformatics approach. Broken arrows indicate that there are multiple biochemical steps involved between the two compounds that connect the identified metabolic intermediates.

Specifically, we found that strain M213 consistently utilized NAP over the 7-day growth period with an increase of cellular biomass (S7 Fig); GC-MS analysis of these samples collected over the 7-day period showed the formation of standard metabolic intermediates of the NAP biodegradative pathway, such as salicylaldehyde and salicylic acid in all the samples; except those that were collected on day 0, which contained only naphthalene (Table 3). Moreover, GC-MS analysis also revealed the presence of additional metabolites to indicate that NAP degradation in M213 branches out via the phthalate pathway. For example, we found 2-carboxy cinnamic acid, 2-hydroxy cinnamic acid, phthalic anhydride, coumarin and hydrocoumarin as metabolic intermediates from NAP over the 7-day growth period, respectively (Table 3). The proposed peculiar pathway for NAP degradation by strain M213 is shown in Fig 7. Formation of 2-carboxy cinnamic acid from 1,2-dihydroxynaphthalene via an ortho-type cleavage (intradiol) mechanism has been previously proposed [13]. Coumarin has also been previously reported as a metabolic intermediate in bacterial metabolism of NAP [13, 64]. In our analysis, coumarin likely resulted via the cyclization of 2-hydroxy cinnamic acid or 2-carboxy cinnamic acid [65] and eventually oxidizing into phthalic anhydride or phthalic acid [13, 65]. To our knowledge, apart from this study, there is only one other report in which both SAL and OPA have been found as metabolic intermediates from the degradation of NAP—by *Rhodococcus ruber* strain OA1 [20]. However, it remains to be understood why strain M213 was unable to grow on salicylate (S2A Fig) but grew vigorously on other metabolic intermediates including OPA (S2C Fig) and protocatechuate (PC) (S2F Fig), respectively. It may be that strain M213 is deficient in the uptake of SAL.

Along with the identification of the above stated metabolic intermediates when strain M213 was grown on NAP, we also located the following genes for NAP degradation to acetyl-CoA and other potential pathway branches, using the metabolic pathway predictor, BioCyc (http://biocyc.org/); parenthesis contains the locus_tag and contig location of the identified genes: Naphthalene dioxygenase large and small subunits (contig 9; WSS_A02450, WSS_A02455), cis-naphthalene dihydrodiol dehydrogenase (contig 9; WSS_A02465), putative aldolase NarC (contig 9; WSS_A02470), Salicyldehyde dehydrogenase (contig 12; WSS_A04660, WSS_A04600, WSS_A04495), salicylate monooxygenase (contig 12; WSS_A04115), salicylyl-CoA 5-hydroxylase (contig 107; WSS_A32785), phthalate 3,4-dioxygenase alpha subunit (contig 35; WSS_A12368), 3,4-dihydroxyphthalate decarboxylase (contig 392; WSS_A43585 and contig 35; WSS_A12338), protocatechuate 3,4-dioxygenase alpha subunit (contig 87; WSS_A28585), protocatechuate 3,4-dioxygenase beta subunit (contig 87; WSS_A28580), 3-carboxy-cis,cis-muconate cycloisomerase (contig 87; WSS_A28590); 4-carboxymuconolactone decarboxylase (contig 211; WSS_A41220, contig 45; WSS_A14784, contig 222; WSS_A41655), 3-oxoadipate enol-lactone hydrolase (contig 87; WSS_A28595), 3-oxoadipate enol-lactonase (contig 63; WSS_A20004), aminomuconate-semialdehyde dehydrogenase (contig 77; WSS_A25505), 4-oxalocrotonate tautomerase (contig 77; WSS_A25450 and WSS_A25495), 4-oxalocrotonate decarboxylase (contig 77; WSS_A25445); gentisate 1,2-dioxygenase (contig 107; WSS_A32775 and contig 12; WSS_A04130), putative catechol 2,3-dioxygenase (contig 9; WSS_A02370, contig 19; WSS_A07264, contig 77; WSS_A25520), 2-hydroxypenta-2,4-dienoate hydratase (contig 152; WSS_A37624, contig 164; WSS_A38827, contig 142; WSS_A36513), putative 4-hydroxy-2-oxovalerate aldolase (contig 9; WSS_A02420), aldehyde dehydrogenase (contig 256; WSS_A42565, contig 438; WSS_A43820, contig 343; WSS_A43245).

It appears that the nature of gene duplication on GEIs in strain M213, and as discussed above, are a common genomic trait in Rhodococcii. For example, the 9.7 Mb genome of *Rhodococcus* jostii RHA1 is distributed over a chromosome and three large linear plasmids: pRHL1 (1100 kb); pRHL2 (450 kb); and pRHL3 (330 kb) (http://www.bcgsc.bc.ca/cgi-bin/rhodococcus/blast_rha1.pl). These plasmids exhibit a significant degree of gene redundancy, such that several oxygenases including at least 6 ring-hydroxylating dioxygenases and 10 cytochrome P450s were identified. It is very likely that such genomic arrangements found in rhodococcii are functional homologues and facilitates adaptation of the host bacterium to polluted niches via catabolizing various hydrocarbons as and when these carbon sources become available to the bacterium. In fact, genomes of at least three other *Rhodococcus* species- *R*. *aetherovorans* I24 (7 Mb), *R*. *erythropolis* strain PR4 (7 Mb) and *Rhodococcus sp*. DK17 (9.1 Mb) reveal a similar pattern of multiple gene homologues [7, 66, 67]; similar to findings reported here for M213. Moreover, our findings reveal that several GEI-encoded catabolic genes are duplicated in strain M213, an attribute similar to strain RHA1 in which catabolic redundancy was linked to its metabolic versatility [9].

Based on several lines of evidences, including genomics, metabolic analyses and bioinformatics, we hypothesize that NDO genes in M213 form a distinct operon, separate from an *o*-phthalate operon, which shares a common ancestor with a previously described *Mycobacterium* system for anthracene metabolism [14]. It appears that NAP metabolism in M213 passes through protocatechuate (PC), and it may be possible that the PC cluster constitutes a third separate operon. Taken together, our data indicates that there may be several distinct genes for NAP degradation in M213 likely originating via reshuffling of genetic modules and genomic rearrangements. In fact, NAP and OPA genes represent an interesting type of gene redundancy because these genes were found duplicated on two separate GEIs in strain M213. Duplicated gene clusters have been previously described in other bacteria, such as the multiple phenol degradation clusters (mhp genes) in *Dechloromonas aromatica* [68] and the two nearly identical copies of phthalate 4,5-dioxygenase found in *Pseudonocardia dioxanivorans* CB1190. A similar situation exists in *Rhodococcus* sp. strain DK17 that possesses three megaplasmids (380-kb pDK1, 330-kb pDK2, and 750-kb pDK3) and the ability to degrade phthalate [66] with duplicate phthalate operons present on plasmids pDK2 and pDK3, respectively. Other examples of duplicated aromatic degradative genes include the chlorophenol degrading gene clusters *tfd* in *Ralstonia eutropha* JMP134 (pJP4) [69], the *Azoarcus evansii* encoded *abm* gene clusters for 2-aminobenzoate metabolism [70], and the *cat* gene clusters for catechol metabolism in *Burkholderia* strain TH2 [71].

In fact, it is well known that biodegradative pathways are mosaics of genes acquired from disparate sources, and genomic analysis shows that this is the case for strain M213. Utilizing proteomics and genomics, it has been recently demonstrated in *Rhodococcus* sp. TFB that naphthalene degradation likely originated via the combination of different catabolic pathways [24] with at least three sets of NAP biodegradative enzymes under separate regulation patterns. Therefore, such cases represent assembly of biodegradation pathways by recruitment of genes originating from different regulons, as might be the case for the peculiar pathway for NAP degradation in strain M213. However, the advantage of such genetic redundancy to the recruiting bacterial host is yet to be completely understood. The most plausible explanation is the ability of the host bacteria to possess better biodegradative capabilities; a trait that can facilitate competence and survival in a polluted environment. In fact, coexistence of two copies of the naphthalene dioxygenase genes has been previously shown to occur in *Pseudomonas* spp. isolated from the Mediterranean [72]. Recruitment of the initial gene for NAP biodegradation followed by recombination likely resulted in duplicated gene copies with hybrid alleles of these genes with relatively small differences in their amino acid sequence but with enhanced biodegradative capability, thereby transferring a selective advantage on the host strains.

To further understand the naphthalene dioxygenase (NDO) gene duplication in strain M213, PFGE resolved chromosomal DNA and two megaplasmids (see S3 Fig) from strain M213 were gel excised. PFGE extracts were then evaluated by PCR amplification of the 16S rRNA genes using 27F-1492R primers (targeting Domain Bacteria) [73] and *R*. *opacus* specific primers- Ro1-Ro2 [74], respectively; the rationale here being that if isolated plasmid replicons are not contaminated by co-migrating chromosomal DNA, then only the PFGE extracted chromosomal fractions will yield positive 16S rRNA gene amplicons. As expected, PCR amplicons of 16S and Ro1-Ro2 were obtained only from the chromosomal DNA fractions confirming that the two plasmid extracts were obtained in purity and did not contain any co-migrating chromosomal DNA (data not shown). When these isolated replicons were separately amplified by the NDO-specific primers, interestingly, we found the presence of both, naphthalene dioxygenase large subunit genes (*narAa* located on contig 9; WSS_A02450) as well as naphthalene dioxygenase small subunit genes (*narAb* located on contig 9; WSS_A02455) across all three replicons of M213 i.e., the chromosome, pNUO1 and pNUO2, indicating that the first enzyme for NAP biodegradation is duplicated across the genome. This is strong evidence that there may be several different gene copies for NAP degradation in strain M213 potentially involved in collectively degrading NAP. It could also be that distinctly different regulons for NAP degradation are present in M213 such that NAP is channeled via the standard SAL pathway, the OPA pathway or both depending on cues that remain completely unknown at this time.

Moreover, phylogenetic analysis of selected catabolic gene sequences obtained from querying the whole genome sequence of strain M213 was also performed. Specifically, phylogenetic affiliation of the large subunit of naphthalene dioxygenase (NDO) (S5A Fig) and phthalate dioxygenase (PD) (S5B Fig) are shown revealing that NDO in strain M213 is taxonomically closest to that present in *Rhodococcus* sp. 124, which degrades NAP via catechol [6]. This analysis also revealed that M213 NDO clusters with a NAP degrading *Gordonia* strain CC-NAPH129-6, thus making it a possibility of the NAP catabolic genes in M213 to be horizontally exchanged from other actinomycetes [75]. Phthalate dioxygenase gene from M213 showed the highest degree of affiliation with *R*. *jostii* strain RHA1 as well as with *Rhodococcus* sp. strains DK17 and TFB, respectively.

### Predictions on the Catabolic Potential of strain M213 and Genomic Location of NAP Degradative Genes

Out of the total protein coding genes in M213 that were associated with KEGG pathways, approximately 394 catabolic genes were identified, which is approximately 8% of its total genome [27]. Some of these genes are similar to those involved in the metabolism of PAHs [naphthalene, phenanthrene, anthracene, benzo(a)pyrene], halogenated aromatics and aromatic hydrocarbons, including pesticides such as DDT and atrazine. A detailed search using NCBI’s genome server and a COG based analysis revealed that M213 harbors a plethora of biodegradative enzymes such as oxygenases, decarboxylases, hydrolases, etc. (Table 2 and S2 Table).

Additionally, using the enzyme abundance profile search in IMG/ER, we surveyed for the presence of catabolic enzymes, which revealed several unique enzymes in strain M213 relative to other sequenced rhodococcii. For example, a survey for the oxygenase revealed the presence of following unique biodegradative enzymes within the genome of strain M213 (locus tags are shown in parenthesis): EC 1.14.12.7—phthalate 4,5-dioxygenase (WSS_RS33765)-; EC:4.1.1.44, 4- carboxymuconolactone decarboxylase (WSS_RS33465); EC 1.14.14.5—alkanesulfonate monoxygenase (WSS_RS19095); EC 1.13.12.16—nitronate monoxygenase (WSS_RS03485 and WSS_RS12850); EC 1.13.11.16–3-carboxyethylcatechol 2,3-dioxygenase (WSS_RS17075); EC 1.14.15.3—alkane 1-monooxygenase (WSS_RS19475 and WSS_RS19655); EC 1.13.11.3—protocatechuate 3,4-dioxygenase (WSS_RS27990 and WSS_RS27995); EC 1.14.13.84–4-hydroxyacetophenone monoxygenase (WSS_RS18265, WSS_RS26615 and WSS_RS02950); EC 1.14.13.2—p-hydroxybenzoate hydrolyase (WSS_RS04020); EC 1.13.11.5—homogentisate 1,2-dioxygenase (WSS_RS02255 and WSS_RS28255); EC 1.13.11.39—biphenyl-2,3-diol 1,2-dioxygenase (WSS_RS07120); EC 1.13.11.1—catechol 1,2-dioxygenase (WSS_RS02300, WSS_RS19570 and WSS_RS36420); EC 1.14.13.50—pentachlorophenol monoxygenase (WSS_RS06110 and WSS_RS09640); EC 1.14.13.25 –soluble methane monooxygenase (WSS_RS38815); EC 1.14.13.1—salicylate hydroxylase (WSS_RS07935); EC 1.14.12.10—benzoate 1,2-dioxygenases (WSS_RS18835, WSS_RS18840 and WSS_RS18845); EC 1.13.11.24—quercetin 2,3-dioxygenase (WSS_RS00100 and WSS_RS13455); and EC 1.13.11.2—catechol 2,3-dioxygenase (WSS_RS24965), respectively. Overall, this analysis revealed a cohort of biodegradative enzymes, several only present in strain M213, and hence are likely previously undescribed with potentially novel biotechnological applications.

We also attempted to further confirm the plasmid-bound nature of catabolic genes of interest in M213 using optical mapping. A detailed analysis of the optical maps confirmed the presence of a ~728kb genomic assembly in M213, which appears to be a full linear element because blunt ends on each end of the assembly were identified- a strong indication of a linear replicon. Interestingly, we found that contig 11, the second largest contig of 244,263 bp in size of the assembled M213 genome (Fig 1C), mapped strongly to the closed, circular megaplasmid based on restriction pattern with the enzyme *NcoI* (Fig 2B). In addition, the PCR amplified *nar*Aa and *nar*Ab gene sequences obtained from strain M213 mapped to the largest megaplasmid- as shown in the optical mapping output and on contig 11 which most likely houses these genes (S6A Fig); this is a short contig (~40 kb) but is long enough to map accurately to the optical map. BLAST analysis also confirmed that contig 11 is a near perfect match with the *nar*A gene from another *Rhodococcus* sp. B2-1 (GQ503241). Furthermore, a 20 kb fragment of contig 11 matches to *Rhodococcus opacus* B4 plasmid pROB02 nearly perfectly, again providing a strong indication that the initial gene(s) for NAP degradation (the narA gene) is encoded on the largest megaplasmid (pNUO1) in M213. These findings mirror a previous study in which three plasmids were identified in *Rhodococcus* sp. strain SAO101 with the nar genes located on the 1.1 Mb megaplasmid [76]. In addition, it was shown for *Rhodococcus* sp. strain P400 to contain four plasmids with the nar genes located on a 180 kb megaplasmid [6], strongly suggesting that catabolic genes can be recruited via horizontal gene transfer mechanisms and their hyper-recombination within the receiving host bacterium may result in peculiar biodegradative pathways- such as for NAP degradation demonstrated in this study.

To identify the presence of OPA genes within the optical map, gene sequence of phthalate 3,4 dioxygenase was obtained by PCR amplification from strain M213; these were mapped onto contig 74 (S6B Fig), an approximately 150 kb fragment homologue to a portion of the second largest optical mapping fragment (~2.2 Mb) and therefore most likely belongs on the chromosomal replicon of M213. Moreover, BLAST analysis showed an approximately 20 kb fragment of contig 74 to be a strong homologue with the chromosome of *R*. *jostii* RHA1, indicating that genes residing on contig 74, including the OPA degrading genes, are most likely chromosomal rather than plasmid-borne. Thus, it is very likely that NAP is degraded jointly by a cohort of genes present in strain M213 on both, plasmid(s) (such as the NDO gene) and the chromosome (such as OPA genes), which represents a peculiar biodegradative genomic assembly.

This peculiar NAP biodegradative pathway was further confirmed by evaluating the gene expression of NAP, OPA and SAL genes in strain M213. Briefly, cell pellets from the experiment described earlier in this study (where metabolic intermediates were detected over a 7-day growth period on 50 ppm of NAP) (Table 3 and S7 Fig), were extracted for the isolation of total RNA from both, the NAP-induced (grown in the presence of naphthalene) and non-induced (glucose grown) cells. The expressions of naphthalene dioxygenase large subunit (narAa), PAH Rieske Iron-Sulfur protein (NDO ISP_NAR_), phthalate dioxygenase large and small subunits (phtAa, phtAb), salicylate monooxygenase (SMO) and M213-specific 16S rRNA gene obtained from induced and non-induced growth conditions were normalized with the 16S rDNA and comparisons were drawn (S7A and S7B Fig). Notably, a 101-bp product containing part of narAa, a 145-bp product of ISP_NAR_, a 133-bp product of phtAa, a 103-bp product of phtAa and a 95-bp product of SMO- all showed conspicuous gene expression patterns when naphthalene was used as the sole carbon source but some also transcribed, albeit at lower levels, when only glucose was the provided carbon source (S7 Fig); this residual biodegradative enzyme turnover observed in the glucose microcosm occurred likely because the seed culture contained NAP. A 132-bp product containing part of 16S rRNA was also equally expressed in naphthalene- or glucose-containing medium. Overall, this analysis revealed that the ribosome abundance, along with the gene expression levels for those genes that are likely to be involved in the peculiar biodegradation of NAP in strain M213, increased relative to the sampling at 1 day (decrease in Ct). The increase in gene expression levels was consistent with cell growth, and paralleled a decrease in naphthalene concentration as evidenced by GCMS analysis (S7A Fig). In the glucose-grown system (S7B Fig), however, gene expression levels for all genes decreased relative to the sampling at 1 day (increase in Ct), indicating the window for highest cellular activity was within the first 24 hours of growth in the presence of glucose. This is strong evidence that transcriptions of genes for both, the SAL and OPA pathway occurred during the degradation of naphthalene by strain M213. Because naphthalene degradative genes appear to be duplicated across the chromosomal and plasmid replicons in strain M213, it is possible that NAP is degraded by a mosaic of genomic elements via dual pathways functioning via the formation of both- salicylate and phthalate, respectively.

In conclusion, using intertwined approaches to include comparative genomics, metabolic and bioinformatic analysis, we provide a broader understanding on the repertoire of catabolic genes, especially for NAP degradation, in a metabolically versatile soil-borne *Rhodococcus opacus* strain M213. Simultaneously, this study facilitates insights into the genome plasticity of a *Rhodococcus* species via the acquisition, loss, and evolution of catabolic genes, an area of research that remains understudied. It appears that the genome of strain M213 is a mosaic of genes potentially recruited from disparate sources via horizontal gene transfer and/or phage attack followed by genetic reshuffling events resulting in functional plasmid(s) or genomic islands. Thus, transposons appear to be significant elements that foster evolution of biodegradative capabilities among the soil-dwelling *Rhodococcus* species, as demonstrated for strain M213. The acquired diverse and stable genetic traits likely contribute towards the successful adaptation and survival of strain M213 within an oligotrophic soil environment, where survival is made possible mainly via catabolic exploitation of fuel-oil associated hydrocarbons. It appears, therefore, that complex hyper-recombination evolutionary events resulted in the accrual of large numbers of multiple catabolic gene copies in strain M213 that are scattered around a large genome encompassing linear plasmids and a chromosome. Finally, the predicted peculiar pathway proposed herein for naphthalene degradation in strain M213, predicted by a combination of genomic, metabolic and bioinformatics approaches, further facilitates our understanding on the physiology, evolution, and ecological fitness of a soil-dwelling NAP-degrading bacteria isolated from a PAH contaminated environment.

## Supporting Information

S1 Fig COG-based comparisons of *R*. *opacus* strain M213 with *Rhodococcus imtechensis* RKJ300 (A); *Rhodococcus wratislaviensis* strain IFP2016 (B); *Rhodococcus jostii* strain RHA1 (C). This comparison was based on 23 COG categories, which are as follows: 1) Not in COGs; 2) General Function Prediction; 3) Transcription; 4) Lipid Transport and Metabolism; 5) Energy Production and Conversion; 6) Amino Acid Transport and Metabolism; 7) Function Unknown; 8) Secondary Metabolites Biosynthesis, Transport and Catabolism; 9) Carbohydrate Transport and Metabolism; 10) Inorganic ion Transport and Metabolism; 11) Coenzyme Transport and Metabolism; 12) Replication, Recombination and Repair; 13) Signal Transduction Mechanism; 14) Translation, Ribosomal Structure and Biogenesis; 15) Cell Wall/Membrane/Envelope Biogenesis; 16) Post-translational Modification, Protein Turnover, Chaperons; 17) Nucleotide Transport and Metabolism; 18) Defense Mechanisms; 19) Cell Cycle Control, Cell Division and Chromosome Partitioning; 20) Intracellular Trafficking, Secretion, and Vesicular Transport; 21) Cell Motility; 22) RNA Processing and Modification; and 23) Chromatin Structure and Dynamics, respectively. Asterisks on top of the bars represent those COGs in M213 that are different by at least 15% amongst the compared strains.
(DOCX)

S2 Fig Growth of *R*. *opacus* strain M213 on NAP (A), SAL (B), OPA (C), 3HBA (D), 4HPA (E), and PC (F). Each substrate was used at a concentration of 0.1% and growth was monitored by measuring the OD taken at every 3 hourly intervals over a 6-day period. Values shown are averages from duplicate measurements.
(DOCX)

S3 Fig Pulsed field gel electrophoresis (PFGE) resolved genomic DNA of *R*. *opacus* strain M213 showing presence of the chromosomal replicon along with the two megaplasmids. Shown are, lane 1: Lambda ladder, 0.05–1 Mb concatemers of phage λcl857Sam7; Lane 2: *Saccharomyces cerevisiae* ladder, 0.022–1.6 Mb; lanes 3–15, strain M213 genomic DNA that were obtained from several different experimental runs.
(DOCX)

S4 Fig An intact prophage region of approximately 42.5 Kb identified from the whole genome sequence of strain M213. The prophage contains 57 coding sequence (CDS) with a GC content of 65.47%.
(DOCX)

S5 Fig Evolutionary relationships shown for the large subunits of naphthalene dioxygenase (narAa) and phthalate 3,4 dioxygenase (phtAa), the two key enzymes potentially engaged in the dual pathway for NAP degradation in strain M213. Shown are A, representing the 468 amino acid long narAa protein (NCBI accession# EKT84394) or B, the 481 amino acid long phtAa protein (NCBI accession# WP_005256004.1), respectively. Minimum homology and alignment length were kept at 5 to run this analysis using AromaDeg. The red circle in each of the cladogram tree represents the protein sequence extracted from the whole genome sequence of M213 using either NCBI or IMG/ER.
(DOCX)

S6 Fig Optical map of strain M213 focusing on NAP and OPA Genes. A, Optical map obtained suggested that contig 11 corresponded to the megaplasmid pNUO1 in strain M213; pNUO1 was shown to contain gene(s) for the initial degradation of naphthalene in strain M213; B, optical map showing mapping of contig 74 containing gene(s) for *o*-phthalate degradation in strain M213 to the chromosomal fraction.
(DOCX)

S7 Fig Shifts in relative gene expression of *Rhodococcus* sp. strain M213 grown in the presence of naphthalene (A) and Glucose (B). Naphthalene (A): Ct values for each gene targeted [ribosomal RNA, naphthalene dioxygenase large subunit (NAP-LSU), naphthalene dioxygenase Rieske-ISP component (Riesk-SUL), phthalate 3,4-dioxygenase alpha subunit (P34D-A), phthalate 3,4-dioxygenase beta subunit (P34D-B), and Salicylate monooxygenase (Sal-MO)] are graphed, with an overlay of bacterial cell density, as assayed by OD_600_, and naphthalene concentration. Cells continued to grow to a maximum OD at approximately 4 days, but never reached cell densities observed when grown with glucose. Conversely, however, gene expression levels for all genes and ribosome abundance increased relative to the sampling at 1 day (decrease in Ct). The increase in gene expression levels was consistent with cell growth, and paralleled a decrease in naphthalene concentration. Glucose (B): Ct values for each gene targeted [ribosomal RNA, naphthalene dioxygenase large subunit (NAP-LSU), naphthalene dioxygenase Rieske-ISP component (Riesk-SUL), phthalate 3,4-dioxygenase alpha subunit (P34D-A), phthalate 3,4-dioxygenase beta subunit (P34D-B), and Salicylate monooxygenase (Sal-MO)] are graphed, with an overlay of bacterial cell density, as assayed by OD_600_. Highly active cells continued to grow to a maximum OD at approximately 3 days. In general, however, gene expression levels for all genes decreased relative to the sampling at 1 day (increase in Ct), indicating the window for highest cellular activity was early when grown in the presence of glucose.
(DOCX)

S1 Table RT-qPCR primer sequences used in this study. Primers and probes shown in the table were obtained by querying the whole genome sequence of strain M213 from the IMG ER annotated genome.
(DOCX)

S2 Table Gene abundances of predominant COG classes found in the genomes of selected *rhodococcus* species relative to strain M213 using the IMG and NCBI annotations. Strain M213 contained high number of COGs for biodegradative enzymes such as dioxygenases and cytochrome P450.
(DOCX)
