# Supplementary material for: Comparative Genomics and Metabolic Analysis Reveals Peculiar Characteristics of Rhodococcus opacus Strain M213 Particularly for Naphthalene Degradation
Source: PLoS One. 2016 Aug 17;11(8):e0161032. doi: 10.1371/journal.pone.0161032 (PMC4988695; doi:10.1371/journal.pone.0161032)
Supplement: S1 Fig — This comparison was based on 23 COG categories, which are as follows: 1) Not in COGs; 2) General Function Prediction; 3) Transcription; 4) Lipid Transport and Metabolism; 5) Energy Production and Conversion; 6) Amino Acid Transport and Metabolism; 7) Function Unknown; 8) Secondary Metabolites Biosynthesis, Transport and Catabolism; 9) Carbohydrate Transport and Metabolism; 10) Inorganic ion Transport and Metabolism; 11) Coenzyme Transport and Metabolism; 12) Replication, Recombination and Repair; 13) Signal Transduction Mechanism; 14) Translation, Ribosomal Structure and Biogenesis; 15) Cell Wall/Membrane/Envelope Biogenesis; 16) Post-translational Modification, Protein Turnover, Chaperons; 17) Nucleotide Transport and Metabolism; 18) Defense Mechanisms; 19) Cell Cycle Control, Cell Division and Chromosome Partitioning; 20) Intracellular Trafficking, Secretion, and Vesicular Transport; 21) Cell Motility; 22) RNA Processing and Modification; and 23) Chromatin Structure and Dynamics, respectively. Asterisks on top of the bars represent those COGs in M213 that are different by at least 15% amongst the compared strains. (DOCX) [file pone.0161032.s001.docx]

**A**

**C**

**B**

1 2 3 4 5 6 7 8 9 10 11 12 13 14 15 16 17 18 19 20 21 22 23

1 2 3 4 5 6 7 8 9 10 11 12 13 14 15 16 17 18 19 20 21 22 23

1 2 3 4 5 6 7 8 9 10 11 12 13 14 15 16 17 18 19 20 21 22 23

COG Associated Gene Counts

Percentage Difference

Percentage Difference

COG Associated Gene Counts

COG Associated Gene Counts

*****

**S1 Fig.** COG-based comparisons of *R*. *opacus* strain M213 with *Rhodococcus imtechensis* RKJ300 (A); *Rhodococcus* *wratislaviensis* strain IFP2016 (B); *Rhodococcus* *jostii* strain RHA1 (C). This comparison was based on 23 COG categories, which are as follows: 1) Not in COGs; 2) General Function Prediction; 3) Transcription; 4) Lipid Transport and Metabolism; 5) Energy Production and Conversion; 6) Amino Acid Transport and Metabolism; 7) Function Unknown; 8) Secondary Metabolites Biosynthesis, Transport and Catabolism; 9) Carbohydrate Transport and Metabolism; 10) Inorganic ion Transport and Metabolism; 11) Coenzyme Transport and Metabolism; 12) Replication, Recombination and Repair; 13) Signal Transduction Mechanism; 14) Translation, Ribosomal Structure and Biogenesis; 15) Cell Wall/Membrane/Envelope Biogenesis; 16) Post-translational Modification, Protein Turnover, Chaperons; 17) Nucleotide Transport and Metabolism; 18) Defense Mechanisms; 19) Cell Cycle Control, Cell Division and Chromosome Partitioning; 20) Intracellular Trafficking, Secretion, and Vesicular Transport; 21) Cell Motility; 22) RNA Processing and Modification; and 23) Chromatin Structure and Dynamics, respectively. Asterisks on top of the bars represent those COGs in M213 that are different by at least 15% amongst the compared strains.
