# Supplementary material for: Comparative Genomics and Metabolic Analysis Reveals Peculiar Characteristics of Rhodococcus opacus Strain M213 Particularly for Naphthalene Degradation
Source: PLoS One. 2016 Aug 17;11(8):e0161032. doi: 10.1371/journal.pone.0161032 (PMC4988695; doi:10.1371/journal.pone.0161032)
Supplement: S2 Fig — Each substrate was used at a concentration of 0.1% and growth was monitored by measuring the OD taken at every 3 hourly intervals over a 6-day period. Values shown are averages from duplicate measurements. (DOCX) [file pone.0161032.s002.docx]

**A**

**B**

**C**

**D**

Growth (OD 600 nm)

**F**

**E**

**S2 Fig.** Growth of *R*. *opacus* strain M213 on NAP (A), SAL (B), OPA (C), 3HBA (D), 4HPA (E), and PC (F). Each substrate was used at a concentration of 0.1% and growth was monitored by measuring the OD taken at every 3 hourly intervals over a 6-day period. Values shown are averages from duplicate measurements.
