# Supplementary material for: Comparative Genomics and Metabolic Analysis Reveals Peculiar Characteristics of Rhodococcus opacus Strain M213 Particularly for Naphthalene Degradation
Source: PLoS One. 2016 Aug 17;11(8):e0161032. doi: 10.1371/journal.pone.0161032 (PMC4988695; doi:10.1371/journal.pone.0161032)
Supplement: S3 Fig — Shown are, lane 1: Lambda ladder, 0.05–1 Mb concatemers of phage λcl857Sam7; Lane 2: Saccharomyces cerevisiae ladder, 0.022–1.6 Mb; lanes 3–15, strain M213 genomic DNA that were obtained from several different experimental runs. (DOCX) [file pone.0161032.s003.docx]

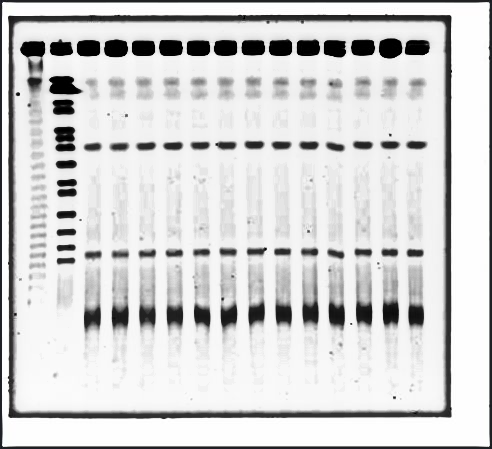


**S3 Fig.** Pulsed field gel electrophoresis (PFGE) resolved genomic DNA of *R. opacus* strain M213 showing presence of the chromosomal replicon along with the two megaplasmids. Shown are, lane 1: Lambda ladder, 0.05-1 Mb concatemers of phage λcl857Sam7; Lane 2: *Saccharomyces cerevisiae* ladder, 0.022-1.6 Mb; lanes 3-15, strain M213 genomic DNA that were obtained from several different experimental runs.

**15**

**14**

**13**

**12**

**11**

**10**

**6**

**7**

**9**

**8**

**4**

**1**

**2**

**3**

**5**

**pNUO2 (350 KB)**

**pNUO1 (750 KB)**

**Chromosomal DNA ‘smear’**
