# Supplementary material for: Comparative Genomics and Metabolic Analysis Reveals Peculiar Characteristics of Rhodococcus opacus Strain M213 Particularly for Naphthalene Degradation
Source: PLoS One. 2016 Aug 17;11(8):e0161032. doi: 10.1371/journal.pone.0161032 (PMC4988695; doi:10.1371/journal.pone.0161032)
Supplement: S4 Fig — The prophage contains 57 coding sequence (CDS) with a GC content of 65.47%. (DOCX) [file pone.0161032.s004.docx]

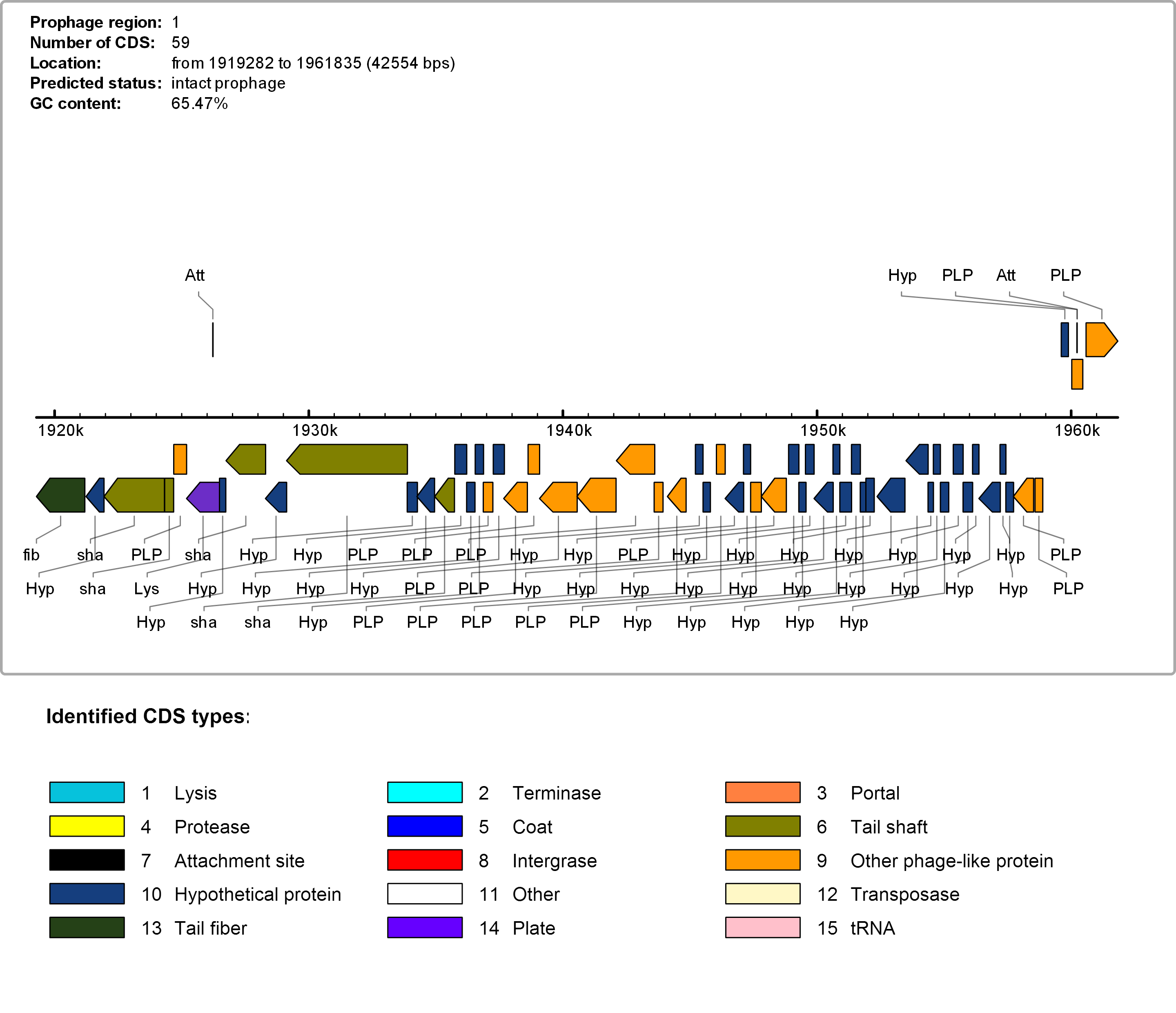


**S4 Fig.** An intact prophage region of approximately 42.5 Kb identified from the whole genome sequence of strain M213. The prophage contains 57 coding sequence (CDS) with a GC content of 65.47%.
