# Supplementary material for: Comparative Genomics and Metabolic Analysis Reveals Peculiar Characteristics of Rhodococcus opacus Strain M213 Particularly for Naphthalene Degradation
Source: PLoS One. 2016 Aug 17;11(8):e0161032. doi: 10.1371/journal.pone.0161032 (PMC4988695; doi:10.1371/journal.pone.0161032)
Supplement: S5 Fig — Shown are A, representing the 468 amino acid long narAa protein (NCBI accession# EKT84394) or B, the 481 amino acid long phtAa protein (NCBI accession# WP_005256004.1), respectively. Minimum homology and alignment length were kept at 5 to run this analysis using AromaDeg. The red circle in each of the cladogram tree represents the protein sequence extracted from the whole genome sequence of M213 using either NCBI or IMG/ER. (DOCX) [file pone.0161032.s005.docx]

***Rhodococcus opacus* M213 Naphthalene Dioxygenase Large Subunit (NDO)**

***Rhodococcus opacus* M213 Phthalate Dioxygenase Large Subunit (phtAa)**

**A**


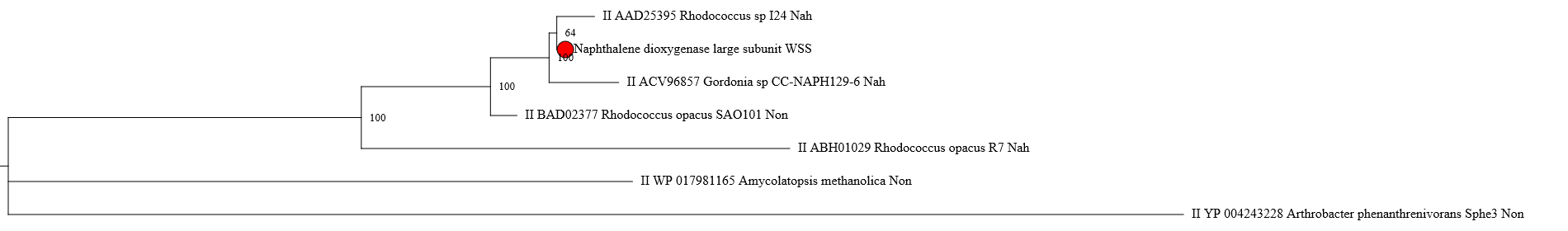

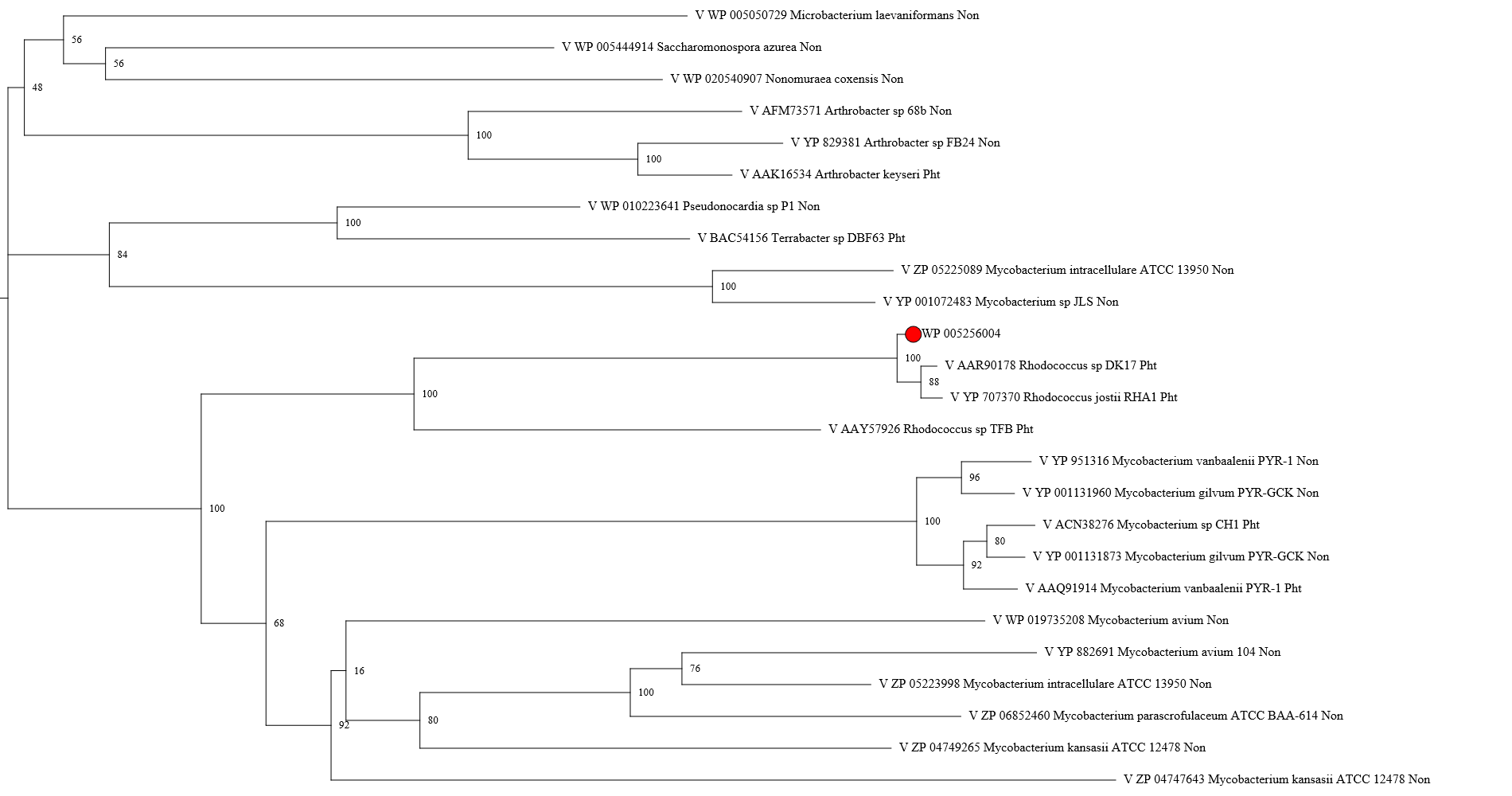


**S5 Fig.** Evolutionary relationships shown for the large subunits of naphthalene dioxygenase (narAa) and phthalate 3,4 dioxygenase (phtAa), the two key enzymes potentially engaged in the dual pathway for NAP degradation in strain M213. The red circle in each of the cladogram tree represents the protein sequence extracted from the whole genome sequence of M213 using either NCBI or IMG/ER. Shown are A, representing the 468 amino acid long narAa protein (NCBI accession# EKT84394) or B, the 481 amino acid long phtAa protein (NCBI accession# WP_005256004.1), respectively. Minimum homology and alignment length were kept at 5 to run this analysis using AromaDeg.
