# Supplementary material for: Comparative Genomics and Metabolic Analysis Reveals Peculiar Characteristics of Rhodococcus opacus Strain M213 Particularly for Naphthalene Degradation
Source: PLoS One. 2016 Aug 17;11(8):e0161032. doi: 10.1371/journal.pone.0161032 (PMC4988695; doi:10.1371/journal.pone.0161032)
Supplement: S6 Fig — A, Optical map obtained suggested that contig 11 corresponded to the megaplasmid pNUO1 in strain M213; pNUO1 was shown to contain gene(s) for the initial degradation of naphthalene in strain M213; B, optical map showing mapping of contig 74 containing gene(s) for o-phthalate degradation in strain M213 to the chromosomal fraction. (DOCX) [file pone.0161032.s006.docx]

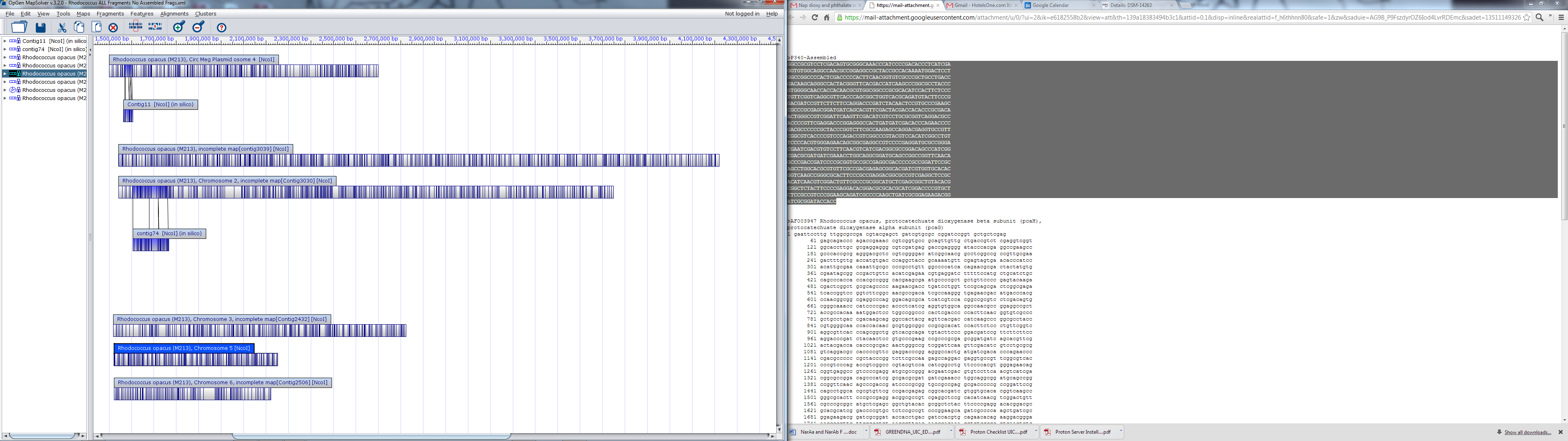


**B**

**A**


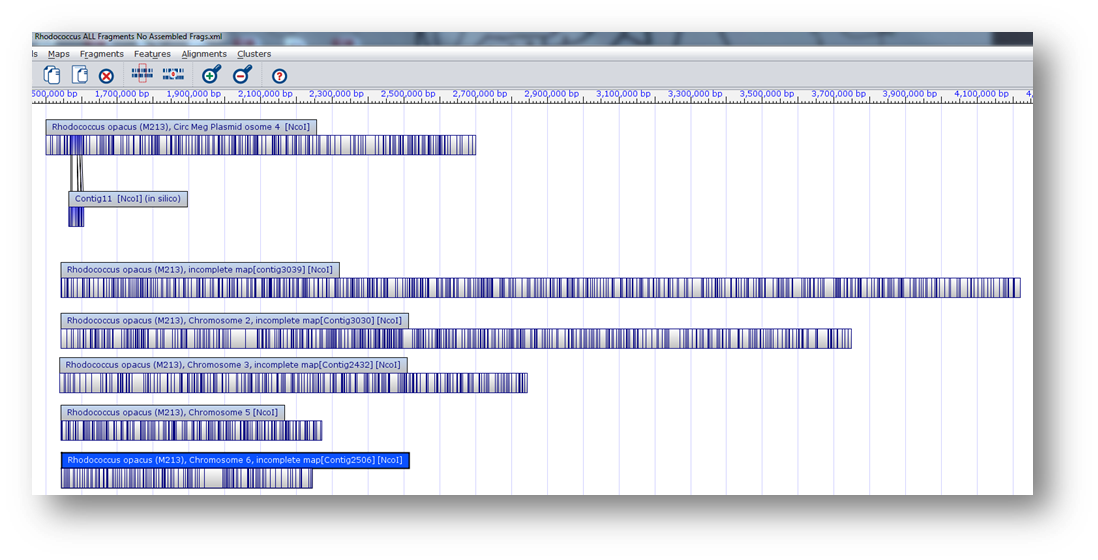


**S6 Fig.** A, Optical map obtained suggested that contig 11 corresponded to the megaplasmid pNUO1 in strain M213; pNUO1 was shown to contain gene(s) for the initial degradation of naphthalene in strain M213; B, optical map showing mapping of contig 74 containing gene(s) for *o*-phthalate degradation in strain M213 to the chromosomal fraction.
