# Supplementary material for: Comparative Genomics and Metabolic Analysis Reveals Peculiar Characteristics of Rhodococcus opacus Strain M213 Particularly for Naphthalene Degradation
Source: PLoS One. 2016 Aug 17;11(8):e0161032. doi: 10.1371/journal.pone.0161032 (PMC4988695; doi:10.1371/journal.pone.0161032)
Supplement: S7 Fig — Naphthalene (A): Ct values for each gene targeted [ribosomal RNA, naphthalene dioxygenase large subunit (NAP-LSU), naphthalene dioxygenase Rieske-ISP component (Riesk-SUL), phthalate 3,4-dioxygenase alpha subunit (P34D-A), phthalate 3,4-dioxygenase beta subunit (P34D-B), and Salicylate monooxygenase (Sal-MO)] are graphed, with an overlay of bacterial cell density, as assayed by OD600, and naphthalene concentration. Cells continued to grow to a maximum OD at approximately 4 days, but never reached cell densities observed when grown with glucose. Conversely, however, gene expression levels for all genes and ribosome abundance increased relative to the sampling at 1 day (decrease in Ct). The increase in gene expression levels was consistent with cell growth, and paralleled a decrease in naphthalene concentration. Glucose (B): Ct values for each gene targeted [ribosomal RNA, naphthalene dioxygenase large subunit (NAP-LSU), naphthalene dioxygenase Rieske-ISP component (Riesk-SUL), phthalate 3,4-dioxygenase alpha subunit (P34D-A), phthalate 3,4-dioxygenase beta subunit (P34D-B), and Salicylate monooxygenase (Sal-MO)] are graphed, with an overlay of bacterial cell density, as assayed by OD600. Highly active cells continued to grow to a maximum OD at approximately 3 days. In general, however, gene expression levels for all genes decreased relative to the sampling at 1 day (increase in Ct), indicating the window for highest cellular activity was early when grown in the presence of glucose. (DOCX) [file pone.0161032.s007.docx]

**A**


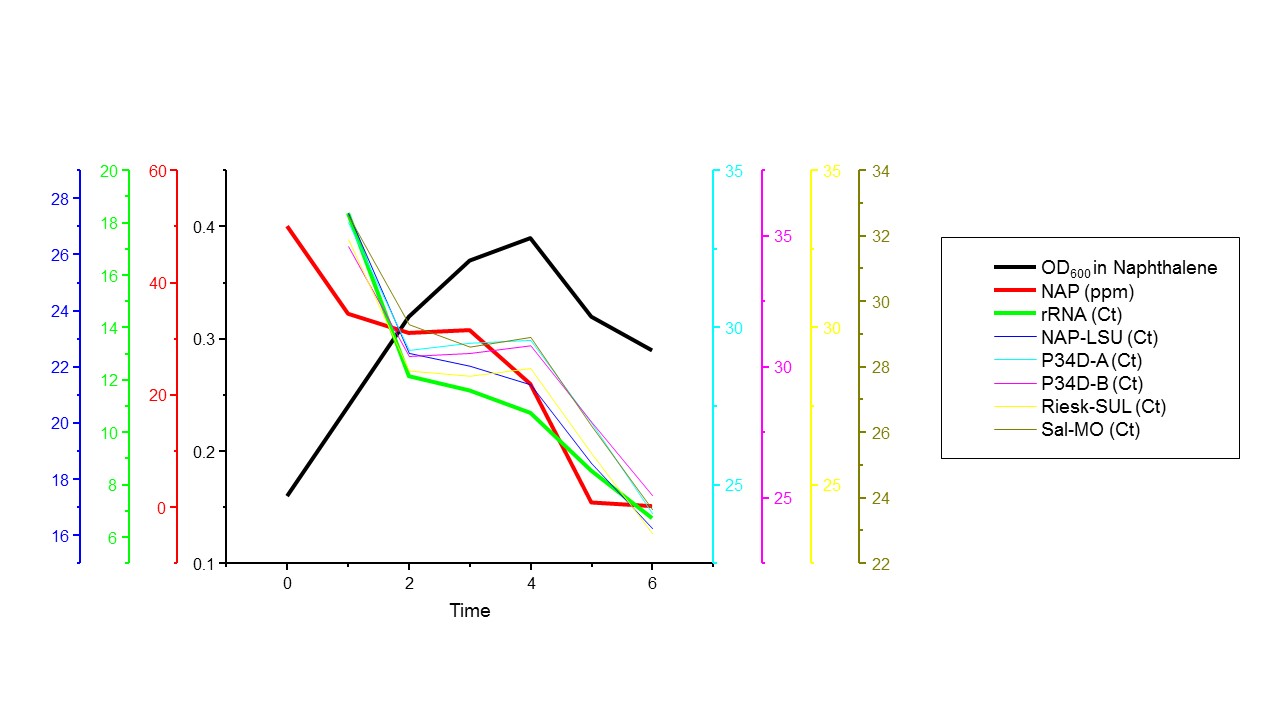


**B**

**S7 Fig.** Shifts in relative gene expression of *Rhodococcus* sp. strain M213 grown in the presence of naphthalene (A). Ct values for each gene targeted [ribosomal RNA, naphthalene dioxygenase large subunit (NAP-LSU), naphthalene dioxygenase Rieske-ISP component (Riesk-SUL), phthalate 3,4-dioxygenase alpha subunit (P34D-A), phthalate 3,4-dioxygenase beta subunit (P34D-B), and Salicylate monooxygenase (Sal-MO)] are graphed, with an overlay of bacterial cell density, as assayed by OD_600_, and naphthalene concentration. Cells continued to grow to a maximum OD at approximately 4 days, but never reached cell densities observed when grown with glucose. Conversely, however, gene expression levels for all genes and ribosome abundance increased relative to the sampling at 1 day (decrease in Ct). The increase in gene expression levels was consistent with cell growth, and paralleled a decrease in naphthalene concentration. Shifts in relative gene expression of *Rhodococcus* sp. strain M213 grown in the presence of glucose (B). Ct values for each gene targeted [ribosomal RNA, naphthalene dioxygenase large subunit (NAP-LSU), naphthalene dioxygenase Rieske-ISP component (Riesk-SUL), phthalate 3,4-dioxygenase alpha subunit (P34D-A), phthalate 3,4-dioxygenase beta subunit (P34D-B), and Salicylate monooxygenase (Sal-MO)] are graphed, with an overlay of bacterial cell density, as assayed by OD_600_. Highly active cells continued to grow to a maximum OD at approximately 3 days. In general, however, gene expression levels for all genes decreased relative to the sampling at 1 day (increase in Ct), indicating the window for highest cellular activity was early when grown in the presence of glucose.
