# Supplementary material for: Comparative Genomics and Metabolic Analysis Reveals Peculiar Characteristics of Rhodococcus opacus Strain M213 Particularly for Naphthalene Degradation
Source: PLoS One. 2016 Aug 17;11(8):e0161032. doi: 10.1371/journal.pone.0161032 (PMC4988695; doi:10.1371/journal.pone.0161032)
Supplement: S1 Table — Primers and probes shown in the table were obtained by querying the whole genome sequence of strain M213 from the IMG ER annotated genome. (DOCX) [file pone.0161032.s008.docx]

**Table S1. RT-qPCR primer sequences used in this study. Primers and probes* were obtained by querying the whole genome sequence of strain M213 from the IMG ER annotated genome.**

**Gene Target IMG ER Oligo Oligo Amplicon**

**Identifier Forward Reverse Size**

NDO (narAa) 2520224645 GTTCAAGGACGAGAGCTACAAG CCAGAACACGGGTGATACTG 102

Rieske ISP 2520228094 GATCAACTGCCACTACCCG TCCTGCTTGAATCCGAGTTC 145

OPA (phtAa) 2520226606 CGTTCATCTCCATTCGACTCTG ATCCGAAGCACATCAGGTATG 133

OPA (phtAb) 2520226605 CCACTACATCACCAACATCCG GACTCATTGACATCCCCTCTG 103

SMO 2520224978 GTGCCACTTCATCCAGTACC CCAGTTCTCGATTCCGTTCTTG 95

16S NA ATGCAAGTCGAGCGGTAAG ATGCAGCCGAAGGTCATATC 130

*Probes ordered at IDT are as follows: NDO:/56-FAM/ATCCCGAAG/Zen/GTGTGCAGGTAGG/3IABkFQ/; Rieske ISP: /56-FAM/CCATGCCGT/Zen/TCGCCACCTC/3IABkFQ/; phtAa: /56-FAM/CAACACCTC/Zen/GGTCTCGTTCTCACTG/3IABkFQ/; phtAb: /56-FAM/AACAGCAAT/Zen/TCGGCGCTTTCGAC/3IABkFQ/; SMO: /56-FAM/ACCAGGTCG/Zen/CGGTGTTCGAAT/3IABkFQ/; 16S: /56-FAM/TTCGGGATA/Zen/AGCCTGGGAAACTGG/3IABkFQ/
