# Supplementary material for: Comparative Genomics and Metabolic Analysis Reveals Peculiar Characteristics of Rhodococcus opacus Strain M213 Particularly for Naphthalene Degradation
Source: PLoS One. 2016 Aug 17;11(8):e0161032. doi: 10.1371/journal.pone.0161032 (PMC4988695; doi:10.1371/journal.pone.0161032)
Supplement: S2 Table — Strain M213 contained high number of COGs for biodegradative enzymes such as dioxygenases and cytochrome P450. (DOCX) [file pone.0161032.s009.docx]

**Table S2.** **Gene abundances of predominant COG classes found in the genomes of selected *rhodococcus* species relative to strain M213 using the IMG and NCBI annotations.** Strain M213 contained high number of COGs for biodegradative enzymes such as dioxygenases and cytochrome P450.

| **Gene**  **Class** | ***R. opacus***  **M213** | ***R. jostii***  **RHA1** | ***R. opacus***  **B4** | ***R. imtechensis***  **RKJ300** | ***R. wratislaviensis* IFP2016** |
| --- | --- | --- | --- | --- | --- |

Transposases and

IS elements 100 96 101 96 195

ABC transporter 100 101 291 253 291

Dioxygenases 90 64 55 64 84

Hydrolases 87 149 167 149 195

Monooxygenases 82 64 42 76 96

Sigma factors 35 37 31 37 43

Cytochrome P450 35 27 39 27 32

Decarboxylases 30 26 26 26 32

Histidine kinases 20 28 37 17 26

Phage integrase 8 8 3 13 8

Chaperones 5 19 9 19 39

Heavy metals 1 1 0 1 2
